# Supplementary material for: Correlates of COVID-19 conspiracy theory beliefs in Japan: A cross-sectional study of 28,175 residents
Source: PLoS One. 2024 Dec 30;19(12):e0310673. doi: 10.1371/journal.pone.0310673 (PMC11684702; doi:10.1371/journal.pone.0310673)
Supplement: S1 Table — (PDF) [file pone.0310673.s001.pdf]

STable 1. Pearson's correlation matrix of three COVID-19 conspiracy belief questions from the Oxford Coronavirus Explanations, Attitudes, and Narratives Survey (OCEANS)

	Question 1	Quetsion 2	Question 3
Question 1: Big Pharma created COVID-19 to profit from the vaccines	1.00	0.78	0.69
Question 2: COVID-19 was created to force everyone to get vaccinated	0.78	1.00	0.72
Question 3: The vaccine will be used to carry out mass sterilisation	0.69	0.72	1.00
