# Supplementary material for: Correlates of COVID-19 conspiracy theory beliefs in Japan: A cross-sectional study of 28,175 residents
Source: PLoS One. 2024 Dec 30;19(12):e0310673. doi: 10.1371/journal.pone.0310673 (PMC11684702; doi:10.1371/journal.pone.0310673)
Supplement: S2 Table — (PDF) [file pone.0310673.s002.pdf]

STable 2. Pearson's correlation matrix of seven general vaccine conspiracy belief questions from the Vaccine Conspiracy Beliefs Scale (VCBS)

	Question 1	Question 2	Question 3	Question 4	Question 5	Question 6	Question 7
Question 1: Vaccine safety data is often fabricated	1.00	0.64	0.69	0.62	0.72	0.65	0.56
Question 2: Immunizing children is harmful and this fact is covered up	0.64	1.00	0.77	0.72	0.69	0.72	0.66
Question 3: Pharmaceutical companies cover up the dangers of vaccines	0.69	0.77	1.00	0.78	0.78	0.79	0.70
Question 4: People are deceived about vaccine efficacy	0.62	0.72	0.78	1.00	0.77	0.84	0.75
Question 5: Vaccine efficacy data is often fabricated	0.72	0.69	0.78	0.77	1.00	0.81	0.70
Question 6: People are deceived about vaccine safety	0.65	0.72	0.79	0.84	0.81	1.00	0.76
Question 7: The government is trying to cover up the link between vaccines and autism	0.56	0.66	0.70	0.75	0.70	0.76	1.00
