# Supplementary material for: Correlates of COVID-19 conspiracy theory beliefs in Japan: A cross-sectional study of 28,175 residents
Source: PLoS One. 2024 Dec 30;19(12):e0310673. doi: 10.1371/journal.pone.0310673 (PMC11684702; doi:10.1371/journal.pone.0310673)
Supplement: S3 Table — (PDF) [file pone.0310673.s003.pdf]

STable 3. Descriptive statistics of the results with sampling weights

Census data source for sample weights

Sampling weights were calculated using the raking method with the R package "anesrake". The maximum weight was set at 5. We matched age, sex, prefecture of residence, marital status, annual household income, and education attainment to census data. Data on age, sex, and prefecture of residence were obtained from the Population Estimates in 2021. Data on marital status and educational attainment were obtained from the Population Census in 2020. Data on annual household income were obtained from the National Survey of Family Income and Expenditure in 2019. The results applying sampling weights are shown in the table below.

Variable	Before weighting	After weighting	Census data
Age			
16 yrs	0.06%	0.30%	1.07%
17 yrs	0.14%	0.71%	1.12%
18 yrs	0.61%	1.15%	1.13%
19 yrs	1.22%	1.20%	1.18%
20 yrs	1.24%	1.25%	1.22%
21 yrs	0.97%	1.27%	1.24%
22 yrs	1.16%	1.27%	1.24%
23 yrs	1.44%	1.30%	1.27%
24 yrs	1.68%	1.30%	1.27%
25 yrs	1.31%	1.30%	1.28%
26 yrs	1.37%	1.32%	1.29%
27 yrs	1.30%	1.31%	1.28%
28 yrs	1.09%	1.28%	1.25%
29 yrs	1.30%	1.29%	1.26%
30 yrs	1.20%	1.28%	1.25%
31 yrs	1.20%	1.31%	1.28%
32 yrs	1.33%	1.33%	1.30%
33 yrs	1.59%	1.37%	1.34%
34 yrs	1.65%	1.40%	1.37%
35 yrs	1.48%	1.42%	1.39%
36 yrs	1.45%	1.47%	1.44%
37 yrs	1.57%	1.52%	1.49%
38 yrs	1.56%	1.54%	1.51%
39 yrs	1.69%	1.54%	1.51%
40 yrs	1.57%	1.56%	1.52%
41 yrs	1.84%	1.62%	1.59%
42 yrs	1.86%	1.66%	1.63%
43 yrs	1.93%	1.73%	1.69%
44 yrs	2.03%	1.77%	1.73%
45 yrs	1.99%	1.85%	1.81%
46 yrs	2.00%	1.93%	1.89%
47 yrs	2.03%	2.04%	1.99%
48 yrs	2.04%	2.07%	2.03%
49 yrs	2.06%	2.04%	1.99%
50 yrs	1.94%	1.98%	1.93%
51 yrs	1.90%	1.91%	1.87%
52 yrs	1.74%	1.88%	1.84%
53 yrs	1.68%	1.84%	1.80%
54 yrs	1.72%	1.83%	1.79%
55 yrs	1.44%	1.43%	1.40%
56 yrs	1.77%	1.76%	1.72%
57 yrs	1.63%	1.65%	1.61%
58 yrs	1.58%	1.60%	1.57%
59 yrs	1.59%	1.55%	1.51%
60 yrs	1.59%	1.52%	1.48%

61 yrs	1.79%	1.52%	1.49%
62 yrs	1.60%	1.55%	1.51%
63 yrs	1.58%	1.50%	1.47%
64 yrs	1.38%	1.46%	1.42%
65 yrs	1.88%	1.52%	1.49%
66 yrs	2.03%	1.57%	1.53%
67 yrs	1.89%	1.56%	1.53%
68 yrs	1.84%	1.65%	1.61%
69 yrs	1.76%	1.73%	1.69%
70 yrs	1.81%	1.82%	1.78%
71 yrs	2.23%	1.94%	1.90%
72 yrs	2.08%	2.11%	2.06%
73 yrs	1.90%	2.07%	2.02%
74 yrs	1.41%	1.94%	1.89%
75 yrs	1.21%	1.19%	1.16%
76 yrs	1.64%	1.26%	1.23%
77 yrs	1.52%	1.51%	1.48%
78 yrs	1.17%	1.44%	1.41%
79 yrs	1.09%	1.45%	1.42%
80 yrs	0.60%	1.37%	1.34%
81 yrs	0.03%	0.16%	1.19%
Sex			
Men	49.23%	49.64%	49.53%
Women	50.77%	50.36%	50.47%
Prefecture of residence			
Hokkaido	4.30%	4.11%	4.13%
Aomori	0.92%	0.93%	0.97%
Iwate	0.81%	0.78%	0.93%
Miyagi	2.13%	1.86%	1.84%
Akita	0.70%	0.75%	0.73%
Yamagata	0.82%	0.84%	0.82%
Fukushima	1.31%	1.47%	1.43%
Ibaraki	1.93%	2.34%	2.29%
Tochigi	1.34%	1.56%	1.54%
Gunma	1.35%	1.57%	1.54%
Saitama	5.82%	5.87%	5.95%
Chiba	5.01%	4.96%	5.07%
Tokyo	12.12%	11.74%	11.51%
Kanagawa	7.74%	7.61%	7.48%
Niigata	1.62%	1.72%	1.71%
Toyama	0.72%	0.80%	0.81%
Ishikawa	0.91%	0.80%	0.89%
Fukui	0.52%	0.61%	0.59%
Yamanashi	0.63%	0.65%	0.64%
Nagano	1.48%	1.57%	1.58%
Gifu	1.46%	1.58%	1.54%
Shizuoka	2.69%	2.85%	2.85%
Aichi	6.65%	6.06%	6.02%
Mie	1.36%	1.24%	1.39%
Shiga	0.98%	1.13%	1.12%
Kyoto	2.11%	2.08%	2.04%
Osaka	7.22%	7.00%	7.07%
Hyogo	4.29%	4.19%	4.30%
Nara	1.15%	0.93%	1.04%
Wakayama	0.60%	0.73%	0.71%
Tottori	0.39%	0.42%	0.42%
Shimane	0.46%	0.52%	0.51%
Okayama	1.51%	1.42%	1.47%
Hiroshima	2.26%	2.23%	2.18%
Yamaguchi	0.95%	1.05%	1.03%
Tokushima	0.59%	0.56%	0.56%
Kagawa	0.81%	0.75%	0.74%
Ehime	1.10%	1.04%	1.03%
Kochi	0.48%	0.54%	0.53%
Fukuoka	4.62%	4.14%	4.04%
Saga	0.57%	0.63%	0.62%
Nagasaki	0.91%	1.01%	1.00%
Kumamoto	1.22%	1.36%	1.33%
Oita	0.77%	0.87%	0.86%
Miyazaki	0.66%	0.83%	0.81%
Kagoshima	1.14%	1.22%	1.20%
Okinawa	0.86%	1.06%	1.13%
Marital status			
Married	60.23%	62.01%	60.58%
Unmarried	29.54%	28.10%	28.90%
Widowed	3.55%	4.26%	4.82%

Divorced	6.68%	5.62%	5.70%
Household size			
1 person	20.98%	29.97%	29.46%
2 persons	34.42%	32.35%	32.52%
3 persons	22.76%	18.66%	18.69%
4 persons	15.43%	12.75%	13.02%
5 persons	4.66%	4.31%	4.37%
6 or more persons	1.74%	1.96%	1.94%
Educational attainment			
Upper Secondary School	1.39%	6.96%	10.37%
Lower Secondary School	27.11%	45.36%	44.31%
Specialised Training College (Post-Secondary Courses), Junior College, and College of Technology	22.34%	18.53%	17.67%
University	44.15%	26.34%	24.99%
Master's or doctor's degrees	5.00%	2.81%	2.65%
Annual household income			
<1 million yen	3.55%	2.00%	2.50%
≥1 to <2 million yen	5.59%	9.70%	9.91%
≥2 to <3 million yen	10.21%	14.17%	14.10%
≥3 to <4 million yen	15.14%	15.28%	15.34%
≥4 to <5 million yen	13.87%	13.02%	13.10%
≥5 to <6 million yen	12.16%	10.35%	10.35%
≥6 to <7 million yen	9.33%	8.62%	8.46%
≥7 to <8 million yen	8.79%	6.64%	6.48%
≥8 to <9 million yen	5.90%	5.01%	4.95%
≥9 to <10 million yen	5.03%	3.90%	3.86%
≥10 to <12 million yen	4.98%	4.95%	4.85%
≥12 to <14 million yen	2.13%	2.61%	2.56%
≥14 to <16 million yen	1.37%	1.42%	1.35%
≥16 to <18 million yen	0.51%	0.80%	0.77%
≥18 to <20 million yen	0.44%	0.49%	0.46%
≥20 million yen	1.01%	1.02%	0.97%
