# Supplementary material for: Correlates of COVID-19 conspiracy theory beliefs in Japan: A cross-sectional study of 28,175 residents
Source: PLoS One. 2024 Dec 30;19(12):e0310673. doi: 10.1371/journal.pone.0310673 (PMC11684702; doi:10.1371/journal.pone.0310673)
Supplement: S4 Table — (PDF) [file pone.0310673.s004.pdf]

STable 4. Full table of basic participant characteristics

Variable	Response participants n=28,175	(%)	Imputed population n=28,175	(%)	Weighted imputed population n=28,175	(%)
**Dependent variable**						
The number of conspiracy theory beliefs regarding COVID-19						
0	21,414	(76.0%)	21,414	(76.0%)	21,297	(75.6%)
1	2,876	(10.2%)	2,876	(10.2%)	2,974	(10.6%)
2	1,830	(6.5%)	1,830	(6.5%)	1,836	(6.5%)
3	2,055	(7.3%)	2,055	(7.3%)	2,068	(7.3%)
**Independent variable**						
The number of conspiracy theory beliefs regarding general vaccines						
0	18,798	(66.7%)	18,798	(66.7%)	18,712	(66.4%)
1	3,021	(10.7%)	3,021	(10.7%)	3,010	(10.7%)
2	2,022	(7.2%)	2,022	(7.2%)	2,067	(7.3%)
3	1,180	(4.2%)	1,180	(4.2%)	1,203	(4.3%)
4	873	(3.1%)	873	(3.1%)	865	(3.1%)
5	772	(2.7%)	772	(2.7%)	768	(2.7%)
6	741	(2.6%)	741	(2.6%)	779	(2.8%)
7	768	(2.7%)	768	(2.7%)	770	(2.7%)
Age (years old)						
16–19	573	(2.0%)	573	(2.0%)	949	(3.4%)
20–24	1,829	(6.5%)	1,829	(6.5%)	1,801	(6.4%)
25–29	1,797	(6.4%)	1,797	(6.4%)	1,834	(6.5%)
30–34	1,963	(7.0%)	1,963	(7.0%)	1,885	(6.7%)
35–39	2,186	(7.8%)	2,186	(7.8%)	2,114	(7.5%)
40–44	2,600	(9.2%)	2,600	(9.2%)	2,350	(8.3%)
45–49	2,854	(10.1%)	2,854	(10.1%)	2,798	(9.9%)
50–54	2,529	(9.0%)	2,529	(9.0%)	2,661	(9.4%)
55–59	2,256	(8.0%)	2,256	(8.0%)	2,250	(8.0%)
60–64	2,234	(7.9%)	2,234	(7.9%)	2,125	(7.5%)
65–69	2,648	(9.4%)	2,648	(9.4%)	2,263	(8.0%)
70–74	2,655	(9.4%)	2,655	(9.4%)	2,781	(9.9%)
75–81	2,051	(7.3%)	2,051	(7.3%)	2,362	(8.4%)
Sex						
Men	13,870	(49.2%)	13,870	(49.2%)	13,986	(49.6%)
Women	14,305	(50.8%)	14,305	(50.8%)	14,189	(50.4%)
Marital status						
Married	16,969	(60.2%)	16,969	(60.2%)	17,472	(62.0%)
Unmarried	8,322	(29.5%)	8,322	(29.5%)	7,918	(28.1%)
Widowed	1,001	(3.6%)	1,001	(3.6%)	1,200	(4.3%)
Divorced	1,883	(6.7%)	1,883	(6.7%)	1,585	(5.6%)
Educational attainment						
Lower Secondary School	392	(1.4%)	392	(1.4%)	1,960	(7.0%)
Upper Secondary School	7,592	(27.1%)	7,638	(27.1%)	12,780	(45.4%)
Specialised Training College (Post-Secondary Courses)	3,203	(11.4%)	3,230	(11.5%)	2,775	(9.9%)
Junior College and College of Technology	3,045	(10.9%)	3,065	(10.9%)	2,447	(8.7%)
University	12,368	(44.2%)	12,440	(44.2%)	7,421	(26.3%)
Master's or doctor's degree	1,409	(5.0%)	1,410	(5.0%)	791	(2.8%)
(Missing)	166					
Employment status						
Regular employee	9,722	(34.5%)	9,722	(34.5%)	9,231	(32.8%)
Temporary employee	4,881	(17.3%)	4,881	(17.3%)	4,976	(17.7%)
Self-employed	1,732	(6.1%)	1,732	(6.1%)	1,659	(5.9%)
Employer	990	(3.5%)	990	(3.5%)	980	(3.5%)
Student	1,267	(4.5%)	1,267	(4.5%)	1,387	(4.9%)
Unemployed or retired	4,636	(16.5%)	4,636	(16.5%)	4,768	(16.9%)
Home maker	4,711	(16.7%)	4,711	(16.7%)	4,938	(17.5%)
Others	236	(0.8%)	236	(0.8%)	235	(0.8%)
Annual household income						
0 to <2 million yen	2,415	(10.9%)	2,575	(9.1%)	3,298	(11.7%)
2 to <4 million yen	5,738	(25.9%)	7,142	(25.3%)	8,298	(29.5%)
4 to <5 million yen	2,868	(13.0%)	3,908	(13.9%)	3,669	(13.0%)
5 to <8 million yen	6,082	(27.5%)	8,530	(30.3%)	7,219	(25.6%)
≥8 million yen	5,024	(22.7%)	6,020	(21.4%)	5,692	(20.2%)
(Missing)	6,048					
Household financial assets						
0 to <1 million yen	3,034	(17.5%)	4,330	(15.4%)	5,144	(18.3%)
1 to <4 million yen	3,779	(21.8%)	6,707	(23.8%)	7,434	(26.4%)
4 to <9 million yen	3,440	(19.9%)	6,594	(23.4%)	6,245	(22.2%)
9 to <20 million yen	3,247	(18.7%)	5,349	(19.0%)	4,931	(17.5%)
≥20 million yen	3,825	(22.1%)	5,195	(18.4%)	4,422	(15.7%)
(Missing)	10,850					
Household indebtedness						
None	15,722	(71.5%)	20,145	(71.5%)	19,914	(70.7%)
>0 to <2 million yen	1,510	(6.9%)	2,005	(7.1%)	2,236	(7.9%)
≥2 million yen	4,771	(21.7%)	6,025	(21.4%)	6,025	(21.4%)
(Missing)	6,172					
Information source for COVID-19: Websites of government agencies						

Not use	15,467	(54.9%)	15,467	(54.9%)	16,182	(57.4%)
Use but distrust	827	(2.9%)	827	(2.9%)	850	(3.0%)
Use and trust	11,881	(42.2%)	11,881	(42.2%)	11,143	(39.5%)
Information source for COVID-19: Websites of research institutions						
Not use	25,179	(89.4%)	25,179	(89.4%)	25,468	(90.4%)
Use but distrust	217	(0.8%)	217	(0.8%)	224	(0.8%)
Use and trust	2,779	(9.9%)	2,779	(9.9%)	2,484	(8.8%)
Information source for COVID-19: Video sharing platforms (e.g. YouTube)						
Not use	23,647	(83.9%)	23,647	(83.9%)	23,534	(83.5%)
Use but distrust	1,229	(4.4%)	1,229	(4.4%)	1,225	(4.3%)
Use and trust	3,299	(11.7%)	3,299	(11.7%)	3,416	(12.1%)
Information source for COVID-19: LINE						
Not use	23,218	(82.4%)	23,218	(82.4%)	23,040	(81.8%)
Use but distrust	845	(3.0%)	845	(3.0%)	862	(3.1%)
Use and trust	4,112	(14.6%)	4,112	(14.6%)	4,273	(15.2%)
Information source for COVID-19: Twitter						
Not use	24,019	(85.2%)	24,019	(85.2%)	24,080	(85.5%)
Use but distrust	1,431	(5.1%)	1,431	(5.1%)	1,348	(4.8%)
Use and trust	2,725	(9.7%)	2,725	(9.7%)	2,747	(9.8%)
Information source for COVID-19: Facebook						
Not use	26,615	(94.5%)	26,615	(94.5%)	26,605	(94.4%)
Use but distrust	461	(1.6%)	461	(1.6%)	455	(1.6%)
Use and trust	1,099	(3.9%)	1,099	(3.9%)	1,115	(4.0%)
Information source for COVID-19: Instagram						
Not use	26,474	(94.0%)	26,474	(94.0%)	26,362	(93.6%)
Use but distrust	493	(1.7%)	493	(1.7%)	518	(1.8%)
Use and trust	1,208	(4.3%)	1,208	(4.3%)	1,296	(4.6%)
Information source for COVID-19: Web news						
Not use	9,828	(34.9%)	9,828	(34.9%)	10,350	(36.7%)
Use but distrust	4,556	(16.2%)	4,556	(16.2%)	4,308	(15.3%)
Use and trust	13,791	(48.9%)	13,791	(48.9%)	13,517	(48.0%)
Information source for COVID-19: Newspapers						
Not use	15,986	(56.7%)	15,986	(56.7%)	16,996	(60.3%)
Use but distrust	829	(2.9%)	829	(2.9%)	772	(2.7%)
Use and trust	11,360	(40.3%)	11,360	(40.3%)	10,407	(36.9%)
Information source for COVID-19: Magazines						
Not use	25,105	(89.1%)	25,105	(89.1%)	25,338	(89.9%)
Use but distrust	560	(2.0%)	560	(2.0%)	512	(1.8%)
Use and trust	2,510	(8.9%)	2,510	(8.9%)	2,325	(8.3%)
Information source for COVID-19: Books						
Not use	25,840	(91.7%)	25,840	(91.7%)	26,140	(92.8%)
Use but distrust	267	(0.9%)	267	(0.9%)	254	(0.9%)
Use and trust	2,068	(7.3%)	2,068	(7.3%)	1,781	(6.3%)
Information source for COVID-19: TV news						
Not use	5,770	(20.5%)	5,770	(20.5%)	6,261	(22.2%)
Use but distrust	2,892	(10.3%)	2,892	(10.3%)	2,778	(9.9%)
Use and trust	19,513	(69.3%)	19,513	(69.3%)	19,136	(67.9%)
Information source for COVID-19: Tabloid TV shows						
Not use	11,502	(40.8%)	11,502	(40.8%)	11,572	(41.1%)
Use but distrust	3,710	(13.2%)	3,710	(13.2%)	3,517	(12.5%)
Use and trust	12,963	(46.0%)	12,963	(46.0%)	13,086	(46.4%)
Trust in the government of Japan						
Distrust	17,596	(62.5%)	17,596	(62.5%)	17,674	(62.7%)
Trust	10,579	(37.5%)	10,579	(37.5%)	10,501	(37.3%)
Trust in the prefectural administration						
Distrust	13,350	(47.4%)	13,350	(47.4%)	13,603	(48.3%)
Trust	14,825	(52.6%)	14,825	(52.6%)	14,572	(51.7%)
Trust in the municipal administration						
Distrust	12,993	(46.1%)	12,993	(46.1%)	13,359	(47.4%)
Trust	15,182	(53.9%)	15,182	(53.9%)	14,816	(52.6%)
Fear of COVID-19						
None	18,059	(64.1%)	18,059	(64.1%)	17,626	(62.6%)
Feeling	10,116	(35.9%)	10,116	(35.9%)	10,549	(37.4%)
Discriminated against related to COVID-19						
Never	26,563	(94.3%)	26,563	(94.3%)	26,516	(94.1%)
Experienced	1,612	(5.7%)	1,612	(5.7%)	1,659	(5.9%)
Medical history of COVID-19						
None	27,663	(98.2%)	27,663	(98.2%)	27,568	(97.8%)
Diagnosed within the past year	260	(0.9%)	260	(0.9%)	348	(1.2%)
Diagnosed before the past year	252	(0.9%)	252	(0.9%)	258	(0.9%)
Medical history of depression						
Never	24,691	(87.6%)	24,691	(87.6%)	24,603	(87.3%)
Former	2,136	(7.6%)	2,136	(7.6%)	2,101	(7.5%)
Current	1,348	(4.8%)	1,348	(4.8%)	1,470	(5.2%)
Medical history of other mental disorders						
Never	25,647	(91.0%)	25,647	(91.0%)	25,527	(90.6%)
Former	1,229	(4.4%)	1,229	(4.4%)	1,317	(4.7%)
Current	1,299	(4.6%)	1,299	(4.6%)	1,331	(4.7%)
