# Supplementary material for: Correlates of COVID-19 conspiracy theory beliefs in Japan: A cross-sectional study of 28,175 residents
Source: PLoS One. 2024 Dec 30;19(12):e0310673. doi: 10.1371/journal.pone.0310673 (PMC11684702; doi:10.1371/journal.pone.0310673)
Supplement: S5 Table — (PDF) [file pone.0310673.s005.pdf]

STable 5. Descriptive statistics of the original categories for three questions on COVID-19 conspiracy beliefs from the Oxford Coronavirus Explanations, Attitudes, and Narratives Survey (OCEANS) after applying sampling weights

	Q1. Big Pharma created COVID-19 to profit from the vaccines (大手製薬会社が、ワクチンで利益を上げるために新型コロナウイルス感染症を作った)		Q2. COVID-19 was created to force everyone to get vaccinated (新型コロナウイルス感染症は、すべての人々にワクチン接種を余儀なくさせるために作られた)		Q3. The vaccine will be used to carry out mass sterilisation (このワクチンを使って、大規模な不妊化を実行しようとしている)	
**Category**	**n**	**%**	**n**	**%**	**n**	**%**
1. Strongly agree (強く賛成する)	1,311	4.7	1,328	4.7	801	2.8
2. Somewhat agree (多少賛成する)	3,617	12.8	3,739	13.3	2,053	7.3
3. Neither agree nor disagree (どちらでもない)	12,312	43.7	12,697	45.1	12,101	42.9
4. Somewhat disagree (多少反対する)	4,045	14.4	3,746	13.3	3,805	13.5
5. Strongly disagree (強く反対する)	6,890	24.5	6,665	23.7	9,416	33.4
