# Supplementary material for: Correlates of COVID-19 conspiracy theory beliefs in Japan: A cross-sectional study of 28,175 residents
Source: PLoS One. 2024 Dec 30;19(12):e0310673. doi: 10.1371/journal.pone.0310673 (PMC11684702; doi:10.1371/journal.pone.0310673)
Supplement: S6 Table — (PDF) [file pone.0310673.s006.pdf]

STable 6. Descriptive statistics of the original categories for seven questions on general vaccine conspiracy beliefs from the Vaccine Conspiracy Beliefs Scale (VCBS) after applying sampling weights

Category	Q1. Vaccine safety data is often fabricated (ワクチンの安全性に関するデータは捏造されることがよくある)		Q2. Immunizing children is harmful and this fact is covered up (小児のワクチン接種は有害であり、この事実は隠蔽されている)		Q3. Pharmaceutical companies cover up the dangers of vaccines (製薬会社はワクチンの危険性を隠している)		Q4. People are deceived about vaccine efficacy (人々はワクチンの効果について騙されている)		Q5. Vaccine efficacy data is often fabricated (ワクチンの効果に関するデータは捏造されることがよくある)		Q6. People are deceived about vaccine safety (人々はワクチンの安全性について騙されている)		Q7. The government is trying to cover up the link between vaccines and autism (政府はワクチンと自閉症の関連を隠そうとしている)	
	n	%	n	%	n	%	n	%	n	%	n	%	n	%
1. Strongly Disagree (強く反対する)	1,786	6.3	2,079	7.4	1,940	6.9	2,501	8.9	1,981	7.0	2,297	8.2	3,168	11.2
2. Disagree (反対する)	2,492	8.8	3,150	11.2	3,123	11.1	3,834	13.6	3,189	11.3	3,714	13.2	3,814	13.5
3. Somewhat Disagree (やや反対する)	2,601	9.2	3,208	11.4	3,333	11.8	3,824	13.6	3,434	12.2	3,605	12.8	3,043	10.8
4. Neutral (どちらでもない)	15,138	53.7	16,716	59.3	15,119	53.7	14,749	52.3	14,537	51.6	14,680	52.1	16,052	57.0
5. Somewhat Agree (やや賛成する)	4,383	15.6	1,998	7.1	3,269	11.6	2,169	7.7	3,652	13.0	2,622	9.3	1,279	4.5
6. Agree (賛成する)	992	3.5	482	1.7	695	2.5	519	1.8	727	2.6	634	2.3	353	1.3
7. Strongly Agree (強く賛成する)	783	2.8	542	1.9	696	2.5	579	2.1	656	2.3	623	2.2	466	1.7
