# Supplementary material for: Correlates of COVID-19 conspiracy theory beliefs in Japan: A cross-sectional study of 28,175 residents
Source: PLoS One. 2024 Dec 30;19(12):e0310673. doi: 10.1371/journal.pone.0310673 (PMC11684702; doi:10.1371/journal.pone.0310673)
Supplement: S7 Table — (PDF) [file pone.0310673.s007.pdf]

STable 7. Full table of the weighted mean of the number of COVID-19 conspiracy beliefs

Variable	The number of conspiracy theory beliefs regarding COVID-19<br>Mean
The number of conspiracy theory beliefs regarding general vaccines	
0	0.41
1	0.48
2	0.45
3	0.45
4	0.56
5	0.50
6	0.75
7	1.12
Age (years old)	
16–19	0.56
20–24	0.53
25–29	0.48
30–34	0.50
35–39	0.45
40–44	0.44
45–49	0.43
50–54	0.39
55–59	0.42
60–64	0.45
65–69	0.44
70–74	0.43
75–81	0.51
Sex	
Men	0.52
Women	0.39
Marital status	
Married	0.46
Unmarried	0.45
Widowed	0.44
Divorced	0.42
Educational attainment	
Lower Secondary School	0.39
Upper Secondary School	0.45
Specialised Training College (Post-Secondary Courses)	0.46
Junior College and College of Technology	0.44
University	0.49
Master's or doctor's degree	0.42
Employment status	
Regular employee	0.51
Temporary employee	0.41
Self-employed	0.44
Employer	0.58
Student	0.52
Unemployed or retired	0.45
Home maker	0.38
Others	0.38
Annual household income	
0 to <2 million yen	0.44
2 to <4 million yen	0.45
4 to <5 million yen	0.42
5 to <8 million yen	0.44
≥8 million yen	0.52
Household financial assets	
0 to <1 million yen	0.41
1 to <4 million yen	0.48
4 to <9 million yen	0.46
9 to <20 million yen	0.50
≥20 million yen	0.42
Household indebtedness	
None	0.45
>0 to <2 million yen	0.47
≥2 million yen	0.48
Information source for COVID-19: Websites of government agencies	
Not use	0.48
Use but distrust	0.66
Use and trust	0.41
Information source for COVID-19: Websites of research institutions	
Not use	0.44
Use but distrust	0.79
Use and trust	0.58
Information source for COVID-19: Video sharing platforms (e.g. YouTube)	

Not use	0.43
Use but distrust	0.50
Use and trust	0.65
Information source for COVID-19: LINE	
Not use	0.43
Use but distrust	0.57
Use and trust	0.56
Information source for COVID-19: Twitter	
Not use	0.44
Use but distrust	0.48
Use and trust	0.60
Information source for COVID-19: Facebook	
Not use	0.44
Use but distrust	0.66
Use and trust	0.77
Information source for COVID-19: Instagram	
Not use	0.44
Use but distrust	0.60
Use and trust	0.74
Information source for COVID-19: Web news	
Not use	0.50
Use but distrust	0.41
Use and trust	0.44
Information source for COVID-19: Newspapers	
Not use	0.45
Use but distrust	0.54
Use and trust	0.46
Information source for COVID-19: Magazines	
Not use	0.44
Use but distrust	0.59
Use and trust	0.64
Information source for COVID-19: Books	
Not use	0.43
Use but distrust	0.82
Use and trust	0.72
Information source for COVID-19: TV news	
Not use	0.54
Use but distrust	0.48
Use and trust	0.43
Information source for COVID-19: Tabloid TV shows	
Not use	0.46
Use but distrust	0.43
Use and trust	0.46
Trust in the government of Japan	
Distrust	0.40
Trust	0.56
Trust in the prefectural administration	
Distrust	0.42
Trust	0.49
Trust in the municipal administration	
Distrust	0.42
Trust	0.49
Fear of COVID-19	
None	0.40
Feeling	0.56
Discriminated against related to COVID-19	
Never	0.44
Experienced	0.68
Medical history of COVID-19	
None	0.45
Diagnosed within the past year	0.77
Diagnosed before the past year	0.91
Medical history of depression	
Never	0.45
Former	0.47
Current	0.54
Medical history of other mental disorders	
Never	0.45
Former	0.46
Current	0.51
