# Supplementary material for: Correlates of COVID-19 conspiracy theory beliefs in Japan: A cross-sectional study of 28,175 residents
Source: PLoS One. 2024 Dec 30;19(12):e0310673. doi: 10.1371/journal.pone.0310673 (PMC11684702; doi:10.1371/journal.pone.0310673)
Supplement: S8 Table — (PDF) [file pone.0310673.s008.pdf]

STable 8. Full table of associations between independent variables and the number of COVID-19 conspiracy beliefs

	Weighted multivariable adjusted model (n=28,175)	
Variable	Beta	95% CI
The number of conspiracy theory beliefs regarding general vaccines		
0 (reference)	—	—
1	0.059	0.017, 0.102
2	0.045	-0.004, 0.094
3	0.040	-0.022, 0.103
4	0.178	0.098, 0.258
5	0.128	0.042, 0.215
6	0.401	0.299, 0.503
7	0.718	0.593, 0.843
Age (years old)		
45–49 (reference)	—	—
16–19	0.140	0.013, 0.266
20–24	0.080	-0.002, 0.163
25–29	0.029	-0.040, 0.098
30–34	0.060	-0.008, 0.128
35–39	0.027	-0.036, 0.090
40–44	0.001	-0.060, 0.061
50–54	-0.025	-0.085, 0.034
55–59	-0.004	-0.066, 0.058
60–64	0.050	-0.015, 0.116
65–69	0.053	-0.014, 0.121
70–74	0.050	-0.016, 0.116
75–81	0.095	0.015, 0.174
Sex		
Men (reference)	—	—
Women	-0.095	-0.130, -0.061
Marital status		
Married (reference)	—	—
Unmarried	-0.054	-0.095, -0.013
Widowed	-0.012	-0.089, 0.064
Divorced	-0.032	-0.096, 0.031
Educational attainment		
Upper Secondary School (reference)	—	—
Lower Secondary School	-0.089	-0.172, -0.006
Specialised Training College (Post-Secondary Courses)	0.018	-0.026, 0.061
Junior College and College of Technology	0.034	-0.008, 0.077
University	0.009	-0.024, 0.042
Master's or doctor's degrees	-0.066	-0.126, -0.006
Employment status		
Regular employee (reference)	—	—
Temporary employee	-0.051	-0.095, -0.007
Self-employed	-0.102	-0.162, -0.042
Employer	0.004	-0.081, 0.089
Student	-0.061	-0.155, 0.034
Unemployed or retired	-0.078	-0.131, -0.025
Home maker	-0.086	-0.135, -0.037
Others	-0.118	-0.248, 0.012
Annual household income		
4 to <5 million yen (reference)	—	—
0 to <2 million yen	0.038	-0.024, 0.101
2 to <4 million yen	0.035	-0.008, 0.078
5 to <8 million yen	0.016	-0.026, 0.058
≥8 million yen	0.069	0.020, 0.117
Household financial assets		
0 to <1 million yen (reference)	—	—
1 to <4 million yen	0.039	-0.006, 0.084
4 to <9 million yen	0.020	-0.026, 0.066
9 to <20 million yen	0.060	0.010, 0.109
≥20 million yen	-0.016	-0.068, 0.036
Household indebtedness		
None (reference)	—	—
>0 to <2 million yen	-0.025	-0.080, 0.030
≥2 million yen	-0.016	-0.052, 0.020
Information source for COVID-19: Websites of government agencies		
Not used (reference)	—	—
Use but distrust	0.083	-0.011, 0.178
Use and trust	-0.109	-0.139, -0.079
Information source for COVID-19: Websites of research institutions		
Not used (reference)	—	—
Use but distrust	0.036	-0.125, 0.198
Use and trust	0.039	-0.016, 0.094

Information source for COVID-19: Video sharing platforms (e.g. YouTube)		
Not used (reference)	—	—
Use but distrust	-0.002	-0.073, 0.070
Use and trust	0.089	0.038, 0.139
Information source for COVID-19: LINE		
Not used (reference)	—	—
Use but distrust	0.064	-0.029, 0.157
Use and trust	0.053	0.009, 0.097
Information source for COVID-19: Twitter		
Not used (reference)	—	—
Use but distrust	-0.002	-0.069, 0.066
Use and trust	0.048	-0.005, 0.102
Information source for COVID-19: Facebook		
Not used (reference)	—	—
Use but distrust	0.042	-0.079, 0.163
Use and trust	0.089	0.000, 0.178
Information source for COVID-19: Instagram		
Not used (reference)	—	—
Use but distrust	-0.012	-0.120, 0.095
Use and trust	0.093	0.008, 0.178
Information source for COVID-19: Web news		
Not used (reference)	—	—
Use but distrust	-0.080	-0.123, -0.037
Use and trust	-0.068	-0.102, -0.034
Information source for COVID-19: Newspapers		
Not used (reference)	—	—
Use but distrust	-0.035	-0.128, 0.057
Use and trust	0.011	-0.023, 0.045
Information source for COVID-19: Magazines		
Not used (reference)	—	—
Use but distrust	0.004	-0.103, 0.111
Use and trust	0.051	-0.007, 0.109
Information source for COVID-19: Books		
Not used (reference)	—	—
Use but distrust	0.137	-0.011, 0.286
Use and trust	0.136	0.068, 0.204
Information source for COVID-19: TV news		
Not used (reference)	—	—
Use but distrust	-0.040	-0.102, 0.022
Use and trust	-0.081	-0.128, -0.035
Information source for COVID-19: Tabloid TV shows		
Not used (reference)	—	—
Use but distrust	0.005	-0.043, 0.053
Use and trust	0.051	0.015, 0.087
Trust in the government of Japan		
Distrust (reference)	—	—
Trust	0.175	0.139, 0.211
Trust in the prefectural administration		
Distrust (reference)	—	—
Trust	0.026	-0.023, 0.074
Trust in the municipal administration		
Distrust (reference)	—	—
Trust	0.025	-0.023, 0.074
Fear of COVID-19		
None (reference)	—	—
Feeling	0.145	0.115, 0.175
Discriminated against related to COVID-19		
Never (reference)	—	—
Experienced	0.088	0.025, 0.151
Medical history of COVID-19		
None (reference)	—	—
Diagnosed within the past year	0.187	0.011, 0.363
Diagnosed before the past year	0.169	0.024, 0.314
Medical history of depression		
Never (reference)	—	—
Former	0.010	-0.042, 0.062
Current	0.050	-0.026, 0.127
Medical history of other mental disorders		
Never (reference)	—	—
Former	-0.041	-0.105, 0.024
Current	0.007	-0.071, 0.085

Linear regression analysis with a robust error variance was used, applying sampling weights and imputation.

A positive coefficient means holding more conspiracy theories while a negative coefficient means holding fewer conspiracy theories.

The fully adjusted model included all independent variables simultaneously.
