# Supplementary material for: Correlates of COVID-19 conspiracy theory beliefs in Japan: A cross-sectional study of 28,175 residents
Source: PLoS One. 2024 Dec 30;19(12):e0310673. doi: 10.1371/journal.pone.0310673 (PMC11684702; doi:10.1371/journal.pone.0310673)
Supplement: S9 Table — (PDF) [file pone.0310673.s009.pdf]

STable 9. Associations between independent variables and COVID-19 vaccine hesitancy (0 = intend, 1 = hesitant) from weighted Poisson regression analyses with a robust error variance after imputation

	Vaccine hesitancy status				Weighted multivariable adjusted model (n=28,175)	
	Intend		Hesitant			
**Variable**	**n**	**(%)**	**n**	**(%)**	**PR**	**95%CI**
The number of conspiracy beliefs about COVID-19						
0	18,190	(85.4%)	3,107	(14.6%)	(reference)	
1	2,579	(86.7%)	395	(13.3%)	0.79	0.69, 0.90
2	1,577	(85.9%)	259	(14.1%)	0.74	0.65, 0.86
3	1,854	(89.7%)	213	(10.3%)	0.59	0.51, 0.69
The number of conspiracy theory beliefs regarding general vaccines						
0	16,883	(90.2%)	1,829	(9.8%)	(reference)	
1	2,621	(87.1%)	389	(12.9%)	1.28	1.12, 1.47
2	1,697	(82.1%)	370	(17.9%)	1.58	1.38, 1.82
3	964	(80.1%)	240	(19.9%)	1.69	1.45, 1.97
4	692	(80.0%)	173	(19.9%)	1.68	1.39, 2.02
5	520	(67.7%)	248	(32.3%)	2.54	2.20, 2.92
6	432	(55.4%)	347	(44.6%)	3.02	2.59, 3.51
7	391	(50.8%)	379	(49.2%)	3.37	2.97, 3.81

PR = prevalence ratio; CI = confidence interval

The adjusted model simultaneously included conspiracy beliefs about COVID-19, general vaccine conspiracy beliefs, sociodemographic variables, information sources for COVID-19, trust in authorities, fear of COVID-19 and others.
