# Supplementary material for: Correlates of COVID-19 conspiracy theory beliefs in Japan: A cross-sectional study of 28,175 residents
Source: PLoS One. 2024 Dec 30;19(12):e0310673. doi: 10.1371/journal.pone.0310673 (PMC11684702; doi:10.1371/journal.pone.0310673)
Supplement: S10 Table — (PDF) [file pone.0310673.s010.pdf]

STable 10. Associations of the interaction term of conspiracy beliefs about COVI-19 and conspiracy beliefs about general vaccines with COVID-19 vaccine hesitancy (0 = intend, 1 = hesitant) from a weighted Poisson regression analysis with a robust error variance after imputation

	**Weighted multivariable adjusted model (n=28,175)**	
	**PR**	**95%CI**
The number of conspiracy beliefs about COVID-19		
0	(reference)	
1	0.60	0.46, 0.78
2	0.43	0.30, 0.62
3	0.28	0.18, 0.45
The number of conspiracy theory beliefs regarding general vaccines		
(Numeric)	1.16	1.14, 1.18
Interaction term		
*One conspiracy belief about COVID-19*Conspiracy theory beliefs regarding general vaccines (Numeric)*	1.09	1.03, 1.15
*Two conspiracy beliefs about COVID-19*Conspiracy theory beliefs regarding general vaccines (Numeric)*	1.13	1.07, 1.20
*Three conspiracy beliefs about COVID-19*Conspiracy theory beliefs regarding general vaccines (Numeric)*	1.17	1.10, 1.25

PR = prevalence ratio; CI = confidence interval

The adjusted model simultaneously included conspiracy beliefs about COVID-19, general vaccine conspiracy beliefs, sociodemographic variables, information sources for COVID-19, trust in authorities, fear of COVID-19 and other.
