# Supplementary material for: Correlates of COVID-19 conspiracy theory beliefs in Japan: A cross-sectional study of 28,175 residents
Source: PLoS One. 2024 Dec 30;19(12):e0310673. doi: 10.1371/journal.pone.0310673 (PMC11684702; doi:10.1371/journal.pone.0310673)
Supplement: Attachment — Submitted filename: Response to Reviewers.docx [file pone.0310673.s011.docx]

**RESPONSES TO REVIEWERS’ COMMENTS**

**Journal requirements**

**1.** Please ensure that your manuscript meets PLOS ONE's style requirements, including those for file naming. The PLOS ONE style templates can be found at

https://journals.plos.org/plosone/s/file?id=wjVg/PLOSOne_formatting_sample_main_body.pdf

and

https://journals.plos.org/plosone/s/file?id=ba62/PLOSOne_formatting_sample_title_authors_affiliations.pdf

**REPLY**

We have modified our manuscript files to adhere to the style requirements of PLOS ONE.

**2.** In the online submission form you indicate that your data is not available for proprietary reasons and have provided a contact point for accessing this data. Please note that your current contact point is a co-author on this manuscript. According to our Data Policy, the contact point must not be an author on the manuscript and must be an institutional contact, ideally not an individual. Please revise your data statement to a non-author institutional point of contact, such as a data access or ethics committee, and send this to us via return email. Please also include contact information for the third party organization, and please include the full citation of where the data can be found.

**REPLY**

We have modified the data statement.

**Availability of data and material**

The data from this study are not stored in a public repository due to the inclusion of personally identifiable or sensitive information. In accordance with Japan’s ethical guidelines, the Research Ethics Committee of the Osaka International Cancer Institute imposed restrictions on data sharing. For any inquiries regarding the data, please contact the JACSIS research office (jacsis_jastis_office@ymail.ne.jp). Details on data availability can be found on the JACSIS website (https://jacsis-study.jp/dug/index. html).

**3.** We note that there is identifying data in the Supporting Information file < 2_Suppl.pdf>. Due to the inclusion of these potentially identifying data, we have removed this file from your file inventory. Prior to sharing human research participant data, authors should consult with an ethics committee to ensure data are shared in accordance with participant consent and all applicable local laws.

Data sharing should never compromise participant privacy. It is therefore not appropriate to publicly share personally identifiable data on human research participants. The following are examples of data that should not be shared:

-Name, initials, physical address

-Ages more specific than whole numbers

-Internet protocol (IP) address

-Specific dates (birth dates, death dates, examination dates, etc.)

-Contact information such as phone number or email address

-Location data

-ID numbers that seem specific (long numbers, include initials, titled “Hospital ID”) rather than random (small numbers in numerical order)

Data that are not directly identifying may also be inappropriate to share, as in combination they can become identifying. For example, data collected from a small group of participants, vulnerable populations, or private groups should not be shared if they involve indirect identifiers (such as sex, ethnicity, location, etc.) that may risk the identification of study participants.

Additional guidance on preparing raw data for publication can be found in our Data Policy (https://journals.plos.org/plosone/s/data-availability#loc-human-research-participant-data-and-other-sensitive-data) and in the following article: http://www.bmj.com/content/340/bmj.c181.long.

Please remove or anonymize all personal information (Ages more specific than whole numbers), ensure that the data shared are in accordance with participant consent, and re-upload a fully anonymized data set. Please note that spreadsheet columns with personal information must be removed and not hidden as all hidden columns will appear in the published file.

**REPLY**

We carefully reviewed the website and paper you provided. These statements address dataset sharing, not tables. Our supplements contain only tables and no information that could identify individuals, even when combining table data. Therefore, our supplements are suitable for publication.

**Response to Reviewer 1**

I understand they have many results, but understanding can be challenging. This article should be shorter and easier to understand. I have the following comments:

**REPLY**

Thank you for your valuable feedback and suggestions. Please find below our point-by-point response to your comments and concerns.

**1.** Language: Better language (letter errors, grammar... etc.) is needed. It must use the Grammarly program to become a better manuscript.

**REPLY**

We have revised the manuscript using the Grammarly carefully.

**2.** Methodology: There are a lot of references. It must reduce references in the methodology

**REPLY**

We have removed some less important references in the methodology.

**3.** The result is a lot of mixing and results found in this manuscript. Most of the data is old, as shown: There are very long tables, and many tables are also in the appendix. It isn't easy to understand these tables.

**REPLY**

We've simplified the results and tables to focus on the most relevant findings. In addition, we removed some less important supplement tables. However, full tables (Tables 1, 2, and 3) are available in the supplementary file for context and transparency.

(Line 216–274, page 9–14)

**Table 1** shows basic participant characteristics (full table in **STable 4**). The median age of respondents was 50, ranging from 16 to 81. The proportion of women was 50.8%. After imputation and weighting, 10.6% (95% confidence interval [CI] 10.1, 11.1) of the participants believed in one COVID-19 conspiracy theory (OCEANS), 6.5% (95%CI 6.1, 6.9) in two, and 7.3% (95%CI 7.0, 7.8) in three. Thus, the prevalence of harboring at least one COVID-19 conspiracy theory was 24.4% (95%CI 23.7, 25.1). The detailed results of the original categories for three questions on COVID-19 conspiracy beliefs are shown in **STable 5**. On the other hand, participants who believed in at least one conspiracy theory regarding general vaccines (VCBS) constituted 33.6% (detailed results are shown in **STable 6**).

**Table 1. Basic characteristics of 28,175 participants**

| **Variable** | **Response participants** | | **Imputed population** | | **Weighted imputed population** | |
| --- | --- | --- | --- | --- | --- | --- |
|  | **n=28,175** | **(%)** | **n=28,175** | **(%)** | **n=28,175** | **(%)** |
| The number of conspiracy theory beliefs regarding COVID‑19 | | | | | | |
| *0* | 21,414 | (76.0%) | 21,414 | (76.0%) | 21,297 | (75.6%) |
| *1* | 2,876 | (10.2%) | 2,876 | (10.2%) | 2,974 | (10.6%) |
| *2* | 1,830 | (6.5%) | 1,830 | (6.5%) | 1,836 | (6.5%) |
| *3* | 2,055 | (7.3%) | 2,055 | (7.3%) | 2,068 | (7.3%) |
| The number of conspiracy theory beliefs regarding general vaccines | | | | | |  |
| *0* | 18,798 | (66.7%) | 18,798 | (66.7%) | 18,712 | (66.4%) |
| *1* | 3,021 | (10.7%) | 3,021 | (10.7%) | 3,010 | (10.7%) |
| *2* | 2,022 | (7.2%) | 2,022 | (7.2%) | 2,067 | (7.3%) |
| *3* | 1,180 | (4.2%) | 1,180 | (4.2%) | 1,203 | (4.3%) |
| *4* | 873 | (3.1%) | 873 | (3.1%) | 865 | (3.1%) |
| *5* | 772 | (2.7%) | 772 | (2.7%) | 768 | (2.7%) |
| *6* | 741 | (2.6%) | 741 | (2.6%) | 779 | (2.8%) |
| *7* | 768 | (2.7%) | 768 | (2.7%) | 770 | (2.7%) |
| Age (years old) | | | | | | |
| *16–19* | 573 | (2.0%) | 573 | (2.0%) | 949 | (3.4%) |
| *20–24* | 1,829 | (6.5%) | 1,829 | (6.5%) | 1,801 | (6.4%) |
| *25–29* | 1,797 | (6.4%) | 1,797 | (6.4%) | 1,834 | (6.5%) |
| *30–34* | 1,963 | (7.0%) | 1,963 | (7.0%) | 1,885 | (6.7%) |
| *35–39* | 2,186 | (7.8%) | 2,186 | (7.8%) | 2,114 | (7.5%) |
| *40–44* | 2,600 | (9.2%) | 2,600 | (9.2%) | 2,350 | (8.3%) |
| *45–49* | 2,854 | (10.1%) | 2,854 | (10.1%) | 2,798 | (9.9%) |
| *50–54* | 2,529 | (9.0%) | 2,529 | (9.0%) | 2,661 | (9.4%) |
| *55–59* | 2,256 | (8.0%) | 2,256 | (8.0%) | 2,250 | (8.0%) |
| *60–64* | 2,234 | (7.9%) | 2,234 | (7.9%) | 2,125 | (7.5%) |
| *65–69* | 2,648 | (9.4%) | 2,648 | (9.4%) | 2,263 | (8.0%) |
| *70–74* | 2,655 | (9.4%) | 2,655 | (9.4%) | 2,781 | (9.9%) |
| *75–81* | 2,051 | (7.3%) | 2,051 | (7.3%) | 2,362 | (8.4%) |
| Sex | | | | | | |
| *Men* | 13,870 | (49.2%) | 13,870 | (49.2%) | 13,986 | (49.6%) |
| *Women* | 14,305 | (50.8%) | 14,305 | (50.8%) | 14,189 | (50.4%) |
| Marital status | | | | | | |
| *Married* | 16,969 | (60.2%) | 16,969 | (60.2%) | 17,472 | (62.0%) |
| *Unmarried* | 8,322 | (29.5%) | 8,322 | (29.5%) | 7,918 | (28.1%) |
| *Widowed* | 1,001 | (3.6%) | 1,001 | (3.6%) | 1,200 | (4.3%) |
| *Divorced* | 1,883 | (6.7%) | 1,883 | (6.7%) | 1,585 | (5.6%) |
| Educational attainment | | | | | | |
| *Lower Secondary School* | 392 | (1.4%) | 392 | (1.4%) | 1,960 | (7.0%) |
| *Upper Secondary School* | 7,592 | (27.1%) | 7,638 | (27.1%) | 12,780 | (45.4%) |
| *Specialised Training College (Post-Secondary Courses)* | 3,203 | (11.4%) | 3,230 | (11.5%) | 2,775 | (9.9%) |
| *Junior College and College of Technology* | 3,045 | (10.9%) | 3,065 | (10.9%) | 2,447 | (8.7%) |
| *University* | 12,368 | (44.2%) | 12,440 | (44.2%) | 7,421 | (26.3%) |
| *Master’s or doctor’s degree* | 1,409 | (5.0%) | 1,410 | (5.0%) | 791 | (2.8%) |
| *(Missing)* | 166 |  |  |  |  |  |
| Employment status | | | | | | |
| *Regular employee* | 9,722 | (34.5%) | 9,722 | (34.5%) | 9,231 | (32.8%) |
| *Temporary employee* | 4,881 | (17.3%) | 4,881 | (17.3%) | 4,976 | (17.7%) |
| *Self-employed* | 1,732 | (6.1%) | 1,732 | (6.1%) | 1,659 | (5.9%) |
| *Employer* | 990 | (3.5%) | 990 | (3.5%) | 980 | (3.5%) |
| *Student* | 1,267 | (4.5%) | 1,267 | (4.5%) | 1,387 | (4.9%) |
| *Unemployed or retired* | 4,636 | (16.5%) | 4,636 | (16.5%) | 4,768 | (16.9%) |
| *Home maker* | 4,711 | (16.7%) | 4,711 | (16.7%) | 4,938 | (17.5%) |
| *Others* | 236 | (0.8%) | 236 | (0.8%) | 235 | (0.8%) |
| Annual household income | | | | | | |
| *0 to <2 million yen* | 2,415 | (10.9%) | 2,575 | (9.1%) | 3,298 | (11.7%) |
| *2 to <4 million yen* | 5,738 | (25.9%) | 7,142 | (25.3%) | 8,298 | (29.5%) |
| *4 to <5 million yen* | 2,868 | (13.0%) | 3,908 | (13.9%) | 3,669 | (13.0%) |
| *5 to <8 million yen* | 6,082 | (27.5%) | 8,530 | (30.3%) | 7,219 | (25.6%) |
| *≥8 million yen* | 5,024 | (22.7%) | 6,020 | (21.4%) | 5,692 | (20.2%) |
| *(Missing)* | 6,048 |  |  |  |  |  |
| Household financial assets | | | | | | |
| *0 to <1 million yen* | 3,034 | (17.5%) | 4,330 | (15.4%) | 5,144 | (18.3%) |
| *1 to <4 million yen* | 3,779 | (21.8%) | 6,707 | (23.8%) | 7,434 | (26.4%) |
| *4 to <9 million yen* | 3,440 | (19.9%) | 6,594 | (23.4%) | 6,245 | (22.2%) |
| *9 to <20 million yen* | 3,247 | (18.7%) | 5,349 | (19.0%) | 4,931 | (17.5%) |
| *≥20 million yen* | 3,825 | (22.1%) | 5,195 | (18.4%) | 4,422 | (15.7%) |
| *(Missing)* | 10,850 |  |  |  |  |  |
| Household indebtedness | | | | | | |
| *None* | 15,722 | (71.5%) | 20,145 | (71.5%) | 19,914 | (70.7%) |
| *>0 to <2 million yen* | 1,510 | (6.9%) | 2,005 | (7.1%) | 2,236 | (7.9%) |
| *≥2 million yen* | 4,771 | (21.7%) | 6,025 | (21.4%) | 6,025 | (21.4%) |
| *(Missing)* | 6,172 |  |  |  |  |  |

Full table is in STable 4.

**Table 2** presents the weighted mean of the number of COVID‑19 conspiracy beliefs (full table in **STable 7**). University graduates exhibited a higher endorsement of COVID‑19 conspiracy beliefs (0.49), and the highest annual household income group also showed higher conspiracy beliefs (0.52). Paradoxically, participants who distrusted the Japanese government endorsed fewer conspiracy beliefs (0.40) compared to those who trusted the government (0.56).

**Table 2. Weighted means of the number of COVID-19 conspiracy beliefs after imputation**

| **Variable** | **Mean number of conspiracy theory beliefs regarding COVID 19** |
| --- | --- |
| The number of conspiracy theory beliefs regarding general vaccines | |
| *0* | 0.41 |
| *1* | 0.48 |
| *2* | 0.45 |
| *3* | 0.45 |
| *4* | 0.56 |
| *5* | 0.50 |
| *6* | 0.75 |
| *7* | 1.12 |
| Educational attainment | |
| *Lower Secondary School* | 0.39 |
| *Upper Secondary School* | 0.45 |
| *Specialised Training College (Post-Secondary Courses)* | 0.46 |
| *Junior College and College of Technology* | 0.44 |
| *University* | 0.49 |
| *Master’s or doctor’s degree* | 0.42 |
| Employment status | |
| *Regular employee* | 0.51 |
| *Temporary employee* | 0.41 |
| *Self-employed* | 0.44 |
| *Employer* | 0.58 |
| *Student* | 0.52 |
| *Unemployed or retired* | 0.45 |
| *Home maker* | 0.38 |
| *Others* | 0.38 |
| Annual household income | |
| *0 to <2 million yen* | 0.44 |
| *2 to <4 million yen* | 0.45 |
| *4 to <5 million yen* | 0.42 |
| *5 to <8 million yen* | 0.44 |
| *≥8 million yen* | 0.52 |
| Household financial assets | |
| *0 to <1 million yen* | 0.41 |
| *1 to <4 million yen* | 0.48 |
| *4 to <9 million yen* | 0.46 |
| *9 to <20 million yen* | 0.50 |
| *≥20 million yen* | 0.42 |
| Household indebtedness | |
| *None* | 0.45 |
| *>0 to <2 million yen* | 0.47 |
| *≥2 million yen* | 0.48 |
| Information source for COVID-19: Websites of government agencies | |
| *Not use* | 0.48 |
| *Use but distrust* | 0.66 |
| *Use and trust* | 0.41 |
| Information source for COVID-19: Video sharing platforms (e.g. YouTube) | |
| *Not use* | 0.43 |
| *Use but distrust* | 0.50 |
| *Use and trust* | 0.65 |
| Information source for COVID-19: LINE | |
| *Not use* | 0.43 |
| *Use but distrust* | 0.57 |
| *Use and trust* | 0.56 |
| Information source for COVID-19: Twitter | |
| *Not use* | 0.44 |
| *Use but distrust* | 0.48 |
| *Use and trust* | 0.60 |
| Information source for COVID-19: Facebook | |
| *Not use* | 0.44 |
| *Use but distrust* | 0.66 |
| *Use and trust* | 0.77 |
| Information source for COVID-19: Instagram | |
| *Not use* | 0.44 |
| *Use but distrust* | 0.60 |
| *Use and trust* | 0.74 |
| Information source for COVID-19: Books | |
| *Not use* | 0.43 |
| *Use but distrust* | 0.82 |
| *Use and trust* | 0.72 |
| Information source for COVID-19: TV news | |
| *Not use* | 0.54 |
| *Use but distrust* | 0.48 |
| *Use and trust* | 0.43 |
| Information source for COVID-19: Tabloid TV shows | |
| *Not use* | 0.46 |
| *Use but distrust* | 0.43 |
| *Use and trust* | 0.46 |
| Trust in the government of Japan | |
| *Distrust* | 0.40 |
| *Trust* | 0.56 |

Full table is in STable 7.

**Table 3** shows the results of multivariable linear regression analysis with sampling weights after imputation (full table in STable 8). Lower secondary education as well as holding a master’s or doctor’s degree were associated with endorsing fewer COVID-19 conspiracy theories compared with upper secondary education (lower secondary education: B -0.089, 95%CI -0.172, -0.006; master’s or doctor’s degree: B -0.066, 95%CI -0.126, -0.006). Temporary employees (B -0.051, 95%CI -0.095, -0.007), self-employed (B -0.102, 95%CI -0.162, -0.042), unemployed or retired (B -0.078, 95%CI -0.131, -0.025), and home makers (B -0.086, 95%CI -0.135, -0.037) held fewer conspiracy theories compared to regular employees. In addition, higher household income and assets were also associated with endorsing more conspiracy theories (annual household income of ≥8 million yen: B 0.069, 95%CI 0.020, 0.117 vs. 4 to <5 million yen; household financial assets of 9 to <20 million yen: B 0.060, 95%CI 0.010, 0.109 vs. 0 to <1 million yen).

As for information sources, participants who used and trusted the websites of government agencies had fewer conspiracy beliefs than those who did not use the websites of government (B -0.109, 95%CI -0.139, -0.079). On the other hand, participants who used and trusted video sharing platforms (e.g. YouTube) (B 0.089, 95%CI 0.038, 0.139), LINE (B 0.053, 95%CI 0.009, 0.097), Facebook (B 0.089, 95%CI 0.000, 0.178), and Instagram (B 0.093, 95%CI 0.008, 0.178) endorsed more conspiracy theories than those who did not use them. Participants who trusted the information from books endorsed more conspiracy beliefs than those who did not use books (B 0.136, 95%CI 0.068, 0.204). Participants who trusted TV news endorsed fewer conspiracy theories (B -0.081, 95%CI -0.128, -0.035 vs. not used), while those who trusted tabloid TV shows held more conspiracy theories (B 0.051, 95%CI 0.015, 0.087 vs. not used).

Trust in authorities was associated with endorsing COVID-19 conspiracy beliefs. Trust in the government of Japan was associated with holding more conspiracy theories (B 0.175, 95%CI 0.139, 0.211), compared to those who distrusted government.

**Table 3. Associations between independent variables and the number of COVID-19 conspiracy beliefs**

|  | **Weighted multivariable adjusted model** | |
| --- | --- | --- |
|  | **(n=28,175)** | |
| **Variable** | **Beta** | **95% confidence interval** |
| The number of conspiracy theory beliefs regarding general vaccines | | |
| *0 (reference)* | — | — |
| *1* | 0.059 | 0.017, 0.102 |
| *2* | 0.045 | -0.004, 0.094 |
| *3* | 0.040 | -0.022, 0.103 |
| *4* | 0.178 | 0.098, 0.258 |
| *5* | 0.128 | 0.042, 0.215 |
| *6* | 0.401 | 0.299, 0.503 |
| *7* | 0.718 | 0.593, 0.843 |
| Educational attainment | |  |
| *Upper Secondary School (reference)* | — | — |
| *Lower Secondary School* | -0.089 | -0.172, -0.006 |
| *Specialised Training College (Post-Secondary Courses)* | 0.018 | -0.026, 0.061 |
| *Junior College and College of Technology* | 0.034 | -0.008, 0.077 |
| *University* | 0.009 | -0.024, 0.042 |
| *Master’s or doctor’s degrees* | -0.066 | -0.126, -0.006 |
| Employment status | |  |
| *Regular employee (reference)* | — | — |
| *Temporary employee* | -0.051 | -0.095, -0.007 |
| *Self-employed* | -0.102 | -0.162, -0.042 |
| *Employer* | 0.004 | -0.081, 0.089 |
| *Student* | -0.061 | -0.155, 0.034 |
| *Unemployed or retired* | -0.078 | -0.131, -0.025 |
| *Home maker* | -0.086 | -0.135, -0.037 |
| *Others* | -0.118 | -0.248, 0.012 |
| Annual household income | | |
| *4 to <5 million yen (reference)* | — | — |
| *0 to <2 million yen* | 0.038 | -0.024, 0.101 |
| *2 to <4 million yen* | 0.035 | -0.008, 0.078 |
| *5 to <8 million yen* | 0.016 | -0.026, 0.058 |
| *≥8 million yen* | 0.069 | 0.020, 0.117 |
| Household financial assets | | |
| *0 to <1 million yen (reference)* | — | — |
| *1 to <4 million yen* | 0.039 | -0.006, 0.084 |
| *4 to <9 million yen* | 0.020 | -0.026, 0.066 |
| *9 to <20 million yen* | 0.060 | 0.010, 0.109 |
| *≥20 million yen* | -0.016 | -0.068, 0.036 |
| Household indebtedness | |  |
| *None (reference)* | — | — |
| *>0 to <2 million yen* | -0.025 | -0.080, 0.030 |
| *≥2 million yen* | -0.016 | -0.052, 0.020 |
| Information source for COVID-19: Websites of government agencies | | |
| *Not used (reference)* | — | — |
| *Use but distrust* | 0.083 | -0.011, 0.178 |
| *Use and trust* | -0.109 | -0.139, -0.079 |
| Information source for COVID-19: Video sharing platforms (e.g. YouTube) | | |
| *Not used (reference)* | — | — |
| *Use but distrust* | -0.002 | -0.073, 0.070 |
| *Use and trust* | 0.089 | 0.038, 0.139 |
| Information source for COVID-19: LINE | | |
| *Not used (reference)* | — | — |
| *Use but distrust* | 0.064 | -0.029, 0.157 |
| *Use and trust* | 0.053 | 0.009, 0.097 |
| Information source for COVID-19: Twitter | | |
| *Not used (reference)* | — | — |
| *Use but distrust* | -0.002 | -0.069, 0.066 |
| *Use and trust* | 0.048 | -0.005, 0.102 |
| Information source for COVID-19: Facebook | | |
| *Not used (reference)* | — | — |
| *Use but distrust* | 0.042 | -0.079, 0.163 |
| *Use and trust* | 0.089 | 0.000, 0.178 |
| Information source for COVID-19: Instagram | | |
| *Not used (reference)* | — | — |
| *Use but distrust* | -0.012 | -0.120, 0.095 |
| *Use and trust* | 0.093 | 0.008, 0.178 |
| Information source for COVID-19: Books | | |
| *Not used (reference)* | — | — |
| *Use but distrust* | 0.137 | -0.011, 0.286 |
| *Use and trust* | 0.136 | 0.068, 0.204 |
| Information source for COVID-19: TV news | | |
| *Not used (reference)* | — | — |
| *Use but distrust* | -0.040 | -0.102, 0.022 |
| *Use and trust* | -0.081 | -0.128, -0.035 |
| Information source for COVID-19: Tabloid TV shows | | |
| *Not used (reference)* | — | — |
| *Use but distrust* | 0.005 | -0.043, 0.053 |
| *Use and trust* | 0.051 | 0.015, 0.087 |
| Trust in the government of Japan | | |
| *Distrust (reference)* | — | — |
| *Trust* | 0.175 | 0.139, 0.211 |

Full table is in STable 8.

Linear regression analysis with a robust error variance was used, applying sampling weights and imputation.

A positive coefficient means holding more conspiracy theories while a negative coefficient means holding fewer conspiracy theories.

The adjusted model included all independent variables simultaneously.

**4.** Discussion: There is no new result to discuss. Most of these results have been published previously. I can't see new results, and this data is old in use.

**REPLY**

We believe that our study provides novel evidence regarding COVID-19 conspiracy beliefs in Japan. Employing sampling weights based on census data, we estimated the prevalence of COVID-19 conspiracy beliefs and, unlike Western studies, found such beliefs to be more prevalent among those with higher socioeconomic status (higher income & wealth, and regular employment). In addition, respondents who distrusted government held relatively fewer COVID-19 conspiracy beliefs. These findings from Japan offer an opportunity to compare with results from Western countries and gain an understanding of COVID-19 conspiracy theories.

**5.** Therefore, based on the issues identified, the manuscript is not currently suitable for publication in this journal. Significant revisions are necessary to address the concerns raised.

It's crucial to condense the results (tables) and enhance the explanation in the discussion and methodology. These improvements are necessary for the manuscript to be considered for publication.

**REPLY**

Thank you for your feedback. We have revised the manuscript entirely, as you suggested.

**Response to Reviewer 2**

Dear Authors and Research Team,

Your team conducted a social science study on the formation and spread of rumors during the COVID-19 pandemic. This research definitely has important social application value, and I believe it should be published.

However, I still have some questions and suggestions for further consideration and revision.

**REPLY**

We appreciate the time and effort that you have invested in reviewing our manuscript. We have responded to all comments carefully, as shown below.

**Major Opinions**

**1.** In the methods section, the "Study Design, Data Sources, and Participants" discusses the survey questionnaire design. Although you have excluded participants who provided fraudulent responses, I would like to know how your questionnaire distinguishes and confirms the nationality of respondents.

**REPLY**

Unfortunately, our questionnaire did not explicitly ask about nationality. Foreign nationals living in Japan are estimated to represent 2.20% of the population.

**2.** This survey was launched on September 27, 2021, and concluded on October 29, 2021. You discuss trust in authorities; however, until October 4, 2021, the authorities were part of the Yoshihide Suga Cabinet, and after that, they belonged to the first Kishida Fumio Cabinet. When designing the survey, did you specify which authorities the participants should consider? If not, I think a more detailed analysis is necessary, as this is very important for the credibility of certain perspectives in the results and discussion.

**REPLY**

We did not specify which authorities the participants should consider. However, we have confirmed that there is no significant difference in the percentage of those trusting the government before 3 October 2021 and after 4 October 2021 (please see the below tables). Therefore, we believe our interpretation can be valid.

|  | **Response date** | | | |
| --- | --- | --- | --- | --- |
|  | Before 2021-10-03 | | After 2021-10-04 | |
| **Trust in the government of Japan** | n | % | n | % |
| Distrusted | 11294 | 62.8 | 6302 | 61.9 |
| Trusted | 6693 | 37.2 | 3886 | 38.1 |

*p-value = 0.121 (Fisher's exact test)*

**The result of the interaction term between response date and trust in the government of Japan from a multivariable linear regression model**

|  | **Weighted multivariable adjusted model** | |
| --- | --- | --- |
|  | **(n=28,175)** | |
| **Variable** | Beta | 95% CI |
| Trust in the government of Japan | | |
| *Distrust (reference)* | — | — |
| *Trust* | 1.39 | 1.25, 1.55 |
| Response date | |  |
| *Before 2021-10-03 (reference)* | — | — |
| *After 2021-10-04* | 0.95 | 0.85, 1.05 |
| Interaction term (Trust in the government of Japan*Response date) | 0.97 | 0.83, 1.13 |

Dependent variable was the number of endorsing COVID-19 conspiracy theories.

The adjusted model included all independent variables simultaneously.

**Minor Opinions**

**3.** On page 15, the statement "A broader range of conspiracy theories needs to be considered, including conspiracy theories about vaccines" is ambiguous, making it difficult to understand the authors' specific position and the measures they believe should be taken. The authors should improve the clarity of this expression.

**REPLY**

We have revised this sentence to enhance readability.

(Line 298–300, page 15)

These findings suggest that different types of conspiracy theories, such as those related to general vaccines and COVID-19, may interact in complex ways to shape health-related behaviour.

**4.** In Table 3, the values and headers labeled "B" and "95 CI" are not aligned.

**REPLY**

We have modified the headers of tables to align them.

**Response to Reviewer 3**

We appreciate your feedback and suggestions. Please see below for a point-by-point response to your comments.

**1.** The introduction sounds quite fragmented. A more cohesive, discursive paragraph is recommended.

Conspiracy believes appeared to have recorded even 42,2%. Add findings from Who Believes in COVID-19 Conspiracy Theories in Croatia? Prevalence and Predictors of Conspiracy Beliefs.

Tonković M, Dumančić F, Jelić M, Čorkalo Biruški D. Front Psychol. 2021 Jun 18;12:643568. doi: 10.3389/fpsyg.2021.643568. eCollection 2021.

Update in light of the recent publications: Factors associated with COVID-19 booster vaccine hesitancy: a nationwide, cross-sectional survey in Japan. Takamatsu A, Honda H, Miwa T, Tabuchi T, Taniguchi K, Shibuya K, Tokuda Y. Public Health. 2023 Oct;223:72-79. doi: 10.1016/j.puhe.2023.07.022. Epub 2023 Aug 22. PMID: 37619504

Effects of Health Literacy in the Fight Against the COVID-19 Infodemic: The Case of Japan.

Cheng JW, Nishikawa M. Health Commun. 2022 Nov;37(12):1520-1533. doi: 10.1080/10410236.2022.2065745. Epub 2022 May 3. PMID: 35505456

A quantitative content analysis of topical characteristics of the online COVID-19 infodemic in the United States and Japan. Seah M, Iwakuma M. BMC Public Health. 2024 Sep 9;24(1):2447. doi: 10.1186/s12889-024-19813-y. PMID: 39251957 Free PMC article.

Exploratory study to characterise the individual types of health literacy and beliefs and their associations with infection prevention behaviours amid the COVID-19 pandemic in Japan: a longitudinal study. Yagihashi M, Murakami M, Kato M, Yamamura A, Miura A, Hirai K. PeerJ. 2024 Feb 22;12:e16905. doi: 10.7717/peerj.16905. eCollection 2024. PMID: 38406277

**REPLY**

We have read the papers you suggested carefully and reconstructed the introduction section referring to them.

(Line 50–97, page 3–4)

The COVID-19 pandemic has presented challenges to global health, not only due to the virus itself, but also due to the rapid spread of misinformation, particularly conspiracy theories [1,2]. Conspiracy theories, defined as “explanations attributing significant events to a small group of powerful actors engaging in officially unacknowledged malevolent activities” [3–7], involve alternative explanations for the emergence of the novel virus, e.g., that the virus was created to reduce the global population, or that pharmaceutical companies manufactured and spread the virus to reap profits [8]. Such COVID-19 conspiracy theories have undermined public health measures, such as social distancing, hand washing, and mask-wearing [9–13].

As in other countries, COVID-19 conspiracy theories have proliferated in Japan. In 2022, for example, a Trump-inspired political party (Sansei tou), which advanced conspiracy theories about COVID-19 (e.g. laboratory origin narrative and big Pharma plot narratives), secured a seat in the House of Councillors [14]. Conspiracy theories appear to be gaining popularity in Japan. However, in Japan, although a previous study estimated the prevalence of conspiracy theories regarding general vaccines [15], the prevalence of COVID-19 conspiracy theories remains unclear.

Previous studies examined the characteristics of individuals endorsing COVID-19 conspiracy beliefs [13]. Based on previous reports, potential determinants of harboring COVID-19 conspiracy beliefs include socioeconomic and social factors, while potential consequences include health behaviours such as vaccine hesitancy and disregard for hygiene guidelines. Among these antecedents, trust has emerged as a key factor influencing the acceptance or rejection of scientific consensus, particularly regarding conspiracy theories. Scientific information is not directly transmitted from scientists, but relayed by governments and national health institutions [12,13]. Distrust in authorities hampers scientific communication. In addition, trust in science and scientists is also essential. An early study identified trust in science as the strongest predictor of rejecting COVID-19 conspiracy theories [16]. Strengthening trust in science may be a key strategy to counter harmful false beliefs.

Socioeconomic status is also a key factor in predicting conspiracy beliefs [12,13]. Individuals with lower educational attainment were more likely to believe COVID-19 conspiracy theories, potentially due to factors such as intuitive thinking, a lower sense of control, and exposure to low-quality information [13,17]. COVID-19 conspiracy theories are abundant on the internet, especially on social media platforms such as YouTube, Instagram, and Twitter (currently known as X). These platforms can amplify conspiracy narratives [1,18] and be one source of conspiracy theories going viral in countries such as Japan [19]. An early study revealed that those relying more on social media (relative to mainstream media) for COVID-19 news had lower health literacy [20] and were more susceptible to misinformation and conspiracy theories [20,21].

The endorsement of conspiracy theory can be contingent upon sociocultural factors [22], and Japan presents a unique context. Studies have found that individuals who rely on traditional media tend to be less inclined to believe in conspiracy theories [23,24]. However, even in traditional media such as TV news and newspapers, there is a history of biased health reporting in Japan. For example, Japan's human papillomavirus vaccination rate is low, due to excessive reports of false side effects by traditional media [25]. This situation might have facilitated distrust in government officials, healthcare professionals, and scientists. Indeed, Japan had relatively low trust in the government in 2020 among OECD countries [26]. The situation might have increased the prevalence of COVID-19 conspiracy beliefs. Therefore, this study aimed to estimate the prevalence of COVID-19 conspiracy beliefs and explore their correlates among 28,175 residents of Japan.

**Methods**

**2.** The methodology is well-detailed, and the tools are clearly explained, making the study replicable.

**REPLY**

Thank you for assessing the methodology section.

**3.** Although the survey involved a significant sample size of 31,000, the article does not specify whether its characteristics were representative of the general population in Japan. Given the correlation with demographic data, the adoption of stratified sampling would have been advisable. Additionally, the article does not specify the distribution of independent variables, which is particularly important, as these could impact the reported prevalence and correlations.

**REPLY**

As you pointed out, the current respondents had different characteristics from the general population in Japan. However, we used sampling weights to achieve national representativeness.

The distribution of independent variables is shown in Tables 1 and 2 (full tables in the supplement). Please see the tables.

(Title)

Correlates of COVID-19 conspiracy theory beliefs in Japan: A cross-sectional study of 28,175 residents

(Line 28–31, page 2)

This study aimed to estimate the prevalence and correlates of COVID-19 conspiracy beliefs using a survey of 28,175 residents of Japan aged 16–81 years old.

**Methods:** A cross-sectional self-administered survey was conducted from September to October 2021.

(Line 111–113, page 5)

This cross-sectional study used a dataset from the Japan COVID-19 and Society Internet Survey (JACSIS) (https://jacsis-study.jp/), which is a web-based self-administered questionnaire survey.

(Line 348–355, page 17–18)

Although recruitment through an internet research agency can limit the generalizability of the findings to the Japanese population, the data include a diverse sample of residents of Japan, including a substantial representation of those with low socioeconomic status. In addition, we employed sampling weights based on census data to improve external validity. Moreover, this study explored numerous potential correlates, enabling a comprehensive assessment of the characteristics of individuals in Japan who endorse COVID-19 conspiracy theories. These strengths can enhance the generalizability of the findings.

(Table 1)

**Table 1. Basic characteristics of 28,175 participants**

| **Variable** | **Response participants** | | **Imputed population** | | **Weighted imputed population** | |
| --- | --- | --- | --- | --- | --- | --- |
|  | **n=28,175** | **(%)** | **n=28,175** | **(%)** | **n=28,175** | **(%)** |
| The number of conspiracy theory beliefs regarding COVID‑19 | | | | | | |
| *0* | 21,414 | (76.0%) | 21,414 | (76.0%) | 21,297 | (75.6%) |
| *1* | 2,876 | (10.2%) | 2,876 | (10.2%) | 2,974 | (10.6%) |
| *2* | 1,830 | (6.5%) | 1,830 | (6.5%) | 1,836 | (6.5%) |
| *3* | 2,055 | (7.3%) | 2,055 | (7.3%) | 2,068 | (7.3%) |
| The number of conspiracy theory beliefs regarding general vaccines | | | | | |  |
| *0* | 18,798 | (66.7%) | 18,798 | (66.7%) | 18,712 | (66.4%) |
| *1* | 3,021 | (10.7%) | 3,021 | (10.7%) | 3,010 | (10.7%) |
| *2* | 2,022 | (7.2%) | 2,022 | (7.2%) | 2,067 | (7.3%) |
| *3* | 1,180 | (4.2%) | 1,180 | (4.2%) | 1,203 | (4.3%) |
| *4* | 873 | (3.1%) | 873 | (3.1%) | 865 | (3.1%) |
| *5* | 772 | (2.7%) | 772 | (2.7%) | 768 | (2.7%) |
| *6* | 741 | (2.6%) | 741 | (2.6%) | 779 | (2.8%) |
| *7* | 768 | (2.7%) | 768 | (2.7%) | 770 | (2.7%) |
| Age (years old) | | | | | | |
| *16–19* | 573 | (2.0%) | 573 | (2.0%) | 949 | (3.4%) |
| *20–24* | 1,829 | (6.5%) | 1,829 | (6.5%) | 1,801 | (6.4%) |
| *25–29* | 1,797 | (6.4%) | 1,797 | (6.4%) | 1,834 | (6.5%) |
| *30–34* | 1,963 | (7.0%) | 1,963 | (7.0%) | 1,885 | (6.7%) |
| *35–39* | 2,186 | (7.8%) | 2,186 | (7.8%) | 2,114 | (7.5%) |
| *40–44* | 2,600 | (9.2%) | 2,600 | (9.2%) | 2,350 | (8.3%) |
| *45–49* | 2,854 | (10.1%) | 2,854 | (10.1%) | 2,798 | (9.9%) |
| *50–54* | 2,529 | (9.0%) | 2,529 | (9.0%) | 2,661 | (9.4%) |
| *55–59* | 2,256 | (8.0%) | 2,256 | (8.0%) | 2,250 | (8.0%) |
| *60–64* | 2,234 | (7.9%) | 2,234 | (7.9%) | 2,125 | (7.5%) |
| *65–69* | 2,648 | (9.4%) | 2,648 | (9.4%) | 2,263 | (8.0%) |
| *70–74* | 2,655 | (9.4%) | 2,655 | (9.4%) | 2,781 | (9.9%) |
| *75–81* | 2,051 | (7.3%) | 2,051 | (7.3%) | 2,362 | (8.4%) |
| Sex | | | | | | |
| *Men* | 13,870 | (49.2%) | 13,870 | (49.2%) | 13,986 | (49.6%) |
| *Women* | 14,305 | (50.8%) | 14,305 | (50.8%) | 14,189 | (50.4%) |
| Marital status | | | | | | |
| *Married* | 16,969 | (60.2%) | 16,969 | (60.2%) | 17,472 | (62.0%) |
| *Unmarried* | 8,322 | (29.5%) | 8,322 | (29.5%) | 7,918 | (28.1%) |
| *Widowed* | 1,001 | (3.6%) | 1,001 | (3.6%) | 1,200 | (4.3%) |
| *Divorced* | 1,883 | (6.7%) | 1,883 | (6.7%) | 1,585 | (5.6%) |
| Educational attainment | | | | | | |
| *Lower Secondary School* | 392 | (1.4%) | 392 | (1.4%) | 1,960 | (7.0%) |
| *Upper Secondary School* | 7,592 | (27.1%) | 7,638 | (27.1%) | 12,780 | (45.4%) |
| *Specialised Training College (Post-Secondary Courses)* | 3,203 | (11.4%) | 3,230 | (11.5%) | 2,775 | (9.9%) |
| *Junior College and College of Technology* | 3,045 | (10.9%) | 3,065 | (10.9%) | 2,447 | (8.7%) |
| *University* | 12,368 | (44.2%) | 12,440 | (44.2%) | 7,421 | (26.3%) |
| *Master’s or doctor’s degree* | 1,409 | (5.0%) | 1,410 | (5.0%) | 791 | (2.8%) |
| *(Missing)* | 166 |  |  |  |  |  |
| Employment status | | | | | | |
| *Regular employee* | 9,722 | (34.5%) | 9,722 | (34.5%) | 9,231 | (32.8%) |
| *Temporary employee* | 4,881 | (17.3%) | 4,881 | (17.3%) | 4,976 | (17.7%) |
| *Self-employed* | 1,732 | (6.1%) | 1,732 | (6.1%) | 1,659 | (5.9%) |
| *Employer* | 990 | (3.5%) | 990 | (3.5%) | 980 | (3.5%) |
| *Student* | 1,267 | (4.5%) | 1,267 | (4.5%) | 1,387 | (4.9%) |
| *Unemployed or retired* | 4,636 | (16.5%) | 4,636 | (16.5%) | 4,768 | (16.9%) |
| *Home maker* | 4,711 | (16.7%) | 4,711 | (16.7%) | 4,938 | (17.5%) |
| *Others* | 236 | (0.8%) | 236 | (0.8%) | 235 | (0.8%) |
| Annual household income | | | | | | |
| *0 to <2 million yen* | 2,415 | (10.9%) | 2,575 | (9.1%) | 3,298 | (11.7%) |
| *2 to <4 million yen* | 5,738 | (25.9%) | 7,142 | (25.3%) | 8,298 | (29.5%) |
| *4 to <5 million yen* | 2,868 | (13.0%) | 3,908 | (13.9%) | 3,669 | (13.0%) |
| *5 to <8 million yen* | 6,082 | (27.5%) | 8,530 | (30.3%) | 7,219 | (25.6%) |
| *≥8 million yen* | 5,024 | (22.7%) | 6,020 | (21.4%) | 5,692 | (20.2%) |
| *(Missing)* | 6,048 |  |  |  |  |  |
| Household financial assets | | | | | | |
| *0 to <1 million yen* | 3,034 | (17.5%) | 4,330 | (15.4%) | 5,144 | (18.3%) |
| *1 to <4 million yen* | 3,779 | (21.8%) | 6,707 | (23.8%) | 7,434 | (26.4%) |
| *4 to <9 million yen* | 3,440 | (19.9%) | 6,594 | (23.4%) | 6,245 | (22.2%) |
| *9 to <20 million yen* | 3,247 | (18.7%) | 5,349 | (19.0%) | 4,931 | (17.5%) |
| *≥20 million yen* | 3,825 | (22.1%) | 5,195 | (18.4%) | 4,422 | (15.7%) |
| *(Missing)* | 10,850 |  |  |  |  |  |
| Household indebtedness | | | | | | |
| *None* | 15,722 | (71.5%) | 20,145 | (71.5%) | 19,914 | (70.7%) |
| *>0 to <2 million yen* | 1,510 | (6.9%) | 2,005 | (7.1%) | 2,236 | (7.9%) |
| *≥2 million yen* | 4,771 | (21.7%) | 6,025 | (21.4%) | 6,025 | (21.4%) |
| *(Missing)* | 6,172 |  |  |  |  |  |

Full table is in STable 4.

(Table 2)

**Table 2. Weighted means of the number of COVID-19 conspiracy beliefs after imputation**

| **Variable** | **Mean number of conspiracy theory beliefs regarding COVID 19** |
| --- | --- |
| The number of conspiracy theory beliefs regarding general vaccines | |
| *0* | 0.41 |
| *1* | 0.48 |
| *2* | 0.45 |
| *3* | 0.45 |
| *4* | 0.56 |
| *5* | 0.50 |
| *6* | 0.75 |
| *7* | 1.12 |
| Educational attainment | |
| *Lower Secondary School* | 0.39 |
| *Upper Secondary School* | 0.45 |
| *Specialised Training College (Post-Secondary Courses)* | 0.46 |
| *Junior College and College of Technology* | 0.44 |
| *University* | 0.49 |
| *Master’s or doctor’s degree* | 0.42 |
| Employment status | |
| *Regular employee* | 0.51 |
| *Temporary employee* | 0.41 |
| *Self-employed* | 0.44 |
| *Employer* | 0.58 |
| *Student* | 0.52 |
| *Unemployed or retired* | 0.45 |
| *Home maker* | 0.38 |
| *Others* | 0.38 |
| Annual household income | |
| *0 to <2 million yen* | 0.44 |
| *2 to <4 million yen* | 0.45 |
| *4 to <5 million yen* | 0.42 |
| *5 to <8 million yen* | 0.44 |
| *≥8 million yen* | 0.52 |
| Household financial assets | |
| *0 to <1 million yen* | 0.41 |
| *1 to <4 million yen* | 0.48 |
| *4 to <9 million yen* | 0.46 |
| *9 to <20 million yen* | 0.50 |
| *≥20 million yen* | 0.42 |
| Household indebtedness | |
| *None* | 0.45 |
| *>0 to <2 million yen* | 0.47 |
| *≥2 million yen* | 0.48 |
| Information source for COVID-19: Websites of government agencies | |
| *Not use* | 0.48 |
| *Use but distrust* | 0.66 |
| *Use and trust* | 0.41 |
| Information source for COVID-19: Video sharing platforms (e.g. YouTube) | |
| *Not use* | 0.43 |
| *Use but distrust* | 0.50 |
| *Use and trust* | 0.65 |
| Information source for COVID-19: LINE | |
| *Not use* | 0.43 |
| *Use but distrust* | 0.57 |
| *Use and trust* | 0.56 |
| Information source for COVID-19: Twitter | |
| *Not use* | 0.44 |
| *Use but distrust* | 0.48 |
| *Use and trust* | 0.60 |
| Information source for COVID-19: Facebook | |
| *Not use* | 0.44 |
| *Use but distrust* | 0.66 |
| *Use and trust* | 0.77 |
| Information source for COVID-19: Instagram | |
| *Not use* | 0.44 |
| *Use but distrust* | 0.60 |
| *Use and trust* | 0.74 |
| Information source for COVID-19: Books | |
| *Not use* | 0.43 |
| *Use but distrust* | 0.82 |
| *Use and trust* | 0.72 |
| Information source for COVID-19: TV news | |
| *Not use* | 0.54 |
| *Use but distrust* | 0.48 |
| *Use and trust* | 0.43 |
| Information source for COVID-19: Tabloid TV shows | |
| *Not use* | 0.46 |
| *Use but distrust* | 0.43 |
| *Use and trust* | 0.46 |
| Trust in the government of Japan | |
| *Distrust* | 0.40 |
| *Trust* | 0.56 |

Full table is in STable 7.

**4.** In general, the article is difficult to read due to the multiple variables involved and the way results are presented, which makes concusions unclear as well.

**REPLY**

In response to Reviewer 1's comment, we have revised the presentation of the results to enhance readability.

(Line 216–274, page 9–14)

**Table 1** shows basic participant characteristics (full table in **STable 4**). The median age of respondents was 50, ranging from 16 to 81. The proportion of women was 50.8%. After imputation and weighting, 10.6% (95% confidence interval [CI] 10.1, 11.1) of the participants believed in one COVID-19 conspiracy theory (OCEANS), 6.5% (95%CI 6.1, 6.9) in two, and 7.3% (95%CI 7.0, 7.8) in three. Thus, the prevalence of harboring at least one COVID-19 conspiracy theory was 24.4% (95%CI 23.7, 25.1). The detailed results of the original categories for three questions on COVID-19 conspiracy beliefs are shown in **STable 5**. On the other hand, participants who believed in at least one conspiracy theory regarding general vaccines (VCBS) constituted 33.6% (detailed results are shown in **STable 6**).

**Table 1. Basic characteristics of 28,175 participants**

| **Variable** | **Response participants** | | **Imputed population** | | **Weighted imputed population** | |
| --- | --- | --- | --- | --- | --- | --- |
|  | **n=28,175** | **(%)** | **n=28,175** | **(%)** | **n=28,175** | **(%)** |
| The number of conspiracy theory beliefs regarding COVID‑19 | | | | | | |
| *0* | 21,414 | (76.0%) | 21,414 | (76.0%) | 21,297 | (75.6%) |
| *1* | 2,876 | (10.2%) | 2,876 | (10.2%) | 2,974 | (10.6%) |
| *2* | 1,830 | (6.5%) | 1,830 | (6.5%) | 1,836 | (6.5%) |
| *3* | 2,055 | (7.3%) | 2,055 | (7.3%) | 2,068 | (7.3%) |
| The number of conspiracy theory beliefs regarding general vaccines | | | | | |  |
| *0* | 18,798 | (66.7%) | 18,798 | (66.7%) | 18,712 | (66.4%) |
| *1* | 3,021 | (10.7%) | 3,021 | (10.7%) | 3,010 | (10.7%) |
| *2* | 2,022 | (7.2%) | 2,022 | (7.2%) | 2,067 | (7.3%) |
| *3* | 1,180 | (4.2%) | 1,180 | (4.2%) | 1,203 | (4.3%) |
| *4* | 873 | (3.1%) | 873 | (3.1%) | 865 | (3.1%) |
| *5* | 772 | (2.7%) | 772 | (2.7%) | 768 | (2.7%) |
| *6* | 741 | (2.6%) | 741 | (2.6%) | 779 | (2.8%) |
| *7* | 768 | (2.7%) | 768 | (2.7%) | 770 | (2.7%) |
| Age (years old) | | | | | | |
| *16–19* | 573 | (2.0%) | 573 | (2.0%) | 949 | (3.4%) |
| *20–24* | 1,829 | (6.5%) | 1,829 | (6.5%) | 1,801 | (6.4%) |
| *25–29* | 1,797 | (6.4%) | 1,797 | (6.4%) | 1,834 | (6.5%) |
| *30–34* | 1,963 | (7.0%) | 1,963 | (7.0%) | 1,885 | (6.7%) |
| *35–39* | 2,186 | (7.8%) | 2,186 | (7.8%) | 2,114 | (7.5%) |
| *40–44* | 2,600 | (9.2%) | 2,600 | (9.2%) | 2,350 | (8.3%) |
| *45–49* | 2,854 | (10.1%) | 2,854 | (10.1%) | 2,798 | (9.9%) |
| *50–54* | 2,529 | (9.0%) | 2,529 | (9.0%) | 2,661 | (9.4%) |
| *55–59* | 2,256 | (8.0%) | 2,256 | (8.0%) | 2,250 | (8.0%) |
| *60–64* | 2,234 | (7.9%) | 2,234 | (7.9%) | 2,125 | (7.5%) |
| *65–69* | 2,648 | (9.4%) | 2,648 | (9.4%) | 2,263 | (8.0%) |
| *70–74* | 2,655 | (9.4%) | 2,655 | (9.4%) | 2,781 | (9.9%) |
| *75–81* | 2,051 | (7.3%) | 2,051 | (7.3%) | 2,362 | (8.4%) |
| Sex | | | | | | |
| *Men* | 13,870 | (49.2%) | 13,870 | (49.2%) | 13,986 | (49.6%) |
| *Women* | 14,305 | (50.8%) | 14,305 | (50.8%) | 14,189 | (50.4%) |
| Marital status | | | | | | |
| *Married* | 16,969 | (60.2%) | 16,969 | (60.2%) | 17,472 | (62.0%) |
| *Unmarried* | 8,322 | (29.5%) | 8,322 | (29.5%) | 7,918 | (28.1%) |
| *Widowed* | 1,001 | (3.6%) | 1,001 | (3.6%) | 1,200 | (4.3%) |
| *Divorced* | 1,883 | (6.7%) | 1,883 | (6.7%) | 1,585 | (5.6%) |
| Educational attainment | | | | | | |
| *Lower Secondary School* | 392 | (1.4%) | 392 | (1.4%) | 1,960 | (7.0%) |
| *Upper Secondary School* | 7,592 | (27.1%) | 7,638 | (27.1%) | 12,780 | (45.4%) |
| *Specialised Training College (Post-Secondary Courses)* | 3,203 | (11.4%) | 3,230 | (11.5%) | 2,775 | (9.9%) |
| *Junior College and College of Technology* | 3,045 | (10.9%) | 3,065 | (10.9%) | 2,447 | (8.7%) |
| *University* | 12,368 | (44.2%) | 12,440 | (44.2%) | 7,421 | (26.3%) |
| *Master’s or doctor’s degree* | 1,409 | (5.0%) | 1,410 | (5.0%) | 791 | (2.8%) |
| *(Missing)* | 166 |  |  |  |  |  |
| Employment status | | | | | | |
| *Regular employee* | 9,722 | (34.5%) | 9,722 | (34.5%) | 9,231 | (32.8%) |
| *Temporary employee* | 4,881 | (17.3%) | 4,881 | (17.3%) | 4,976 | (17.7%) |
| *Self-employed* | 1,732 | (6.1%) | 1,732 | (6.1%) | 1,659 | (5.9%) |
| *Employer* | 990 | (3.5%) | 990 | (3.5%) | 980 | (3.5%) |
| *Student* | 1,267 | (4.5%) | 1,267 | (4.5%) | 1,387 | (4.9%) |
| *Unemployed or retired* | 4,636 | (16.5%) | 4,636 | (16.5%) | 4,768 | (16.9%) |
| *Home maker* | 4,711 | (16.7%) | 4,711 | (16.7%) | 4,938 | (17.5%) |
| *Others* | 236 | (0.8%) | 236 | (0.8%) | 235 | (0.8%) |
| Annual household income | | | | | | |
| *0 to <2 million yen* | 2,415 | (10.9%) | 2,575 | (9.1%) | 3,298 | (11.7%) |
| *2 to <4 million yen* | 5,738 | (25.9%) | 7,142 | (25.3%) | 8,298 | (29.5%) |
| *4 to <5 million yen* | 2,868 | (13.0%) | 3,908 | (13.9%) | 3,669 | (13.0%) |
| *5 to <8 million yen* | 6,082 | (27.5%) | 8,530 | (30.3%) | 7,219 | (25.6%) |
| *≥8 million yen* | 5,024 | (22.7%) | 6,020 | (21.4%) | 5,692 | (20.2%) |
| *(Missing)* | 6,048 |  |  |  |  |  |
| Household financial assets | | | | | | |
| *0 to <1 million yen* | 3,034 | (17.5%) | 4,330 | (15.4%) | 5,144 | (18.3%) |
| *1 to <4 million yen* | 3,779 | (21.8%) | 6,707 | (23.8%) | 7,434 | (26.4%) |
| *4 to <9 million yen* | 3,440 | (19.9%) | 6,594 | (23.4%) | 6,245 | (22.2%) |
| *9 to <20 million yen* | 3,247 | (18.7%) | 5,349 | (19.0%) | 4,931 | (17.5%) |
| *≥20 million yen* | 3,825 | (22.1%) | 5,195 | (18.4%) | 4,422 | (15.7%) |
| *(Missing)* | 10,850 |  |  |  |  |  |
| Household indebtedness | | | | | | |
| *None* | 15,722 | (71.5%) | 20,145 | (71.5%) | 19,914 | (70.7%) |
| *>0 to <2 million yen* | 1,510 | (6.9%) | 2,005 | (7.1%) | 2,236 | (7.9%) |
| *≥2 million yen* | 4,771 | (21.7%) | 6,025 | (21.4%) | 6,025 | (21.4%) |
| *(Missing)* | 6,172 |  |  |  |  |  |

Full table is in STable 4.

**Table 2** presents the weighted mean of the number of COVID‑19 conspiracy beliefs (full table in **STable 7**). University graduates exhibited a higher endorsement of COVID‑19 conspiracy beliefs (0.49), and the highest annual household income group also showed higher conspiracy beliefs (0.52). Paradoxically, participants who distrusted the Japanese government endorsed fewer conspiracy beliefs (0.40) compared to those who trusted the government (0.56).

**Table 2. Weighted means of the number of COVID-19 conspiracy beliefs after imputation**

| **Variable** | **Mean number of conspiracy theory beliefs regarding COVID 19** |
| --- | --- |
| The number of conspiracy theory beliefs regarding general vaccines | |
| *0* | 0.41 |
| *1* | 0.48 |
| *2* | 0.45 |
| *3* | 0.45 |
| *4* | 0.56 |
| *5* | 0.50 |
| *6* | 0.75 |
| *7* | 1.12 |
| Educational attainment | |
| *Lower Secondary School* | 0.39 |
| *Upper Secondary School* | 0.45 |
| *Specialised Training College (Post-Secondary Courses)* | 0.46 |
| *Junior College and College of Technology* | 0.44 |
| *University* | 0.49 |
| *Master’s or doctor’s degree* | 0.42 |
| Employment status | |
| *Regular employee* | 0.51 |
| *Temporary employee* | 0.41 |
| *Self-employed* | 0.44 |
| *Employer* | 0.58 |
| *Student* | 0.52 |
| *Unemployed or retired* | 0.45 |
| *Home maker* | 0.38 |
| *Others* | 0.38 |
| Annual household income | |
| *0 to <2 million yen* | 0.44 |
| *2 to <4 million yen* | 0.45 |
| *4 to <5 million yen* | 0.42 |
| *5 to <8 million yen* | 0.44 |
| *≥8 million yen* | 0.52 |
| Household financial assets | |
| *0 to <1 million yen* | 0.41 |
| *1 to <4 million yen* | 0.48 |
| *4 to <9 million yen* | 0.46 |
| *9 to <20 million yen* | 0.50 |
| *≥20 million yen* | 0.42 |
| Household indebtedness | |
| *None* | 0.45 |
| *>0 to <2 million yen* | 0.47 |
| *≥2 million yen* | 0.48 |
| Information source for COVID-19: Websites of government agencies | |
| *Not use* | 0.48 |
| *Use but distrust* | 0.66 |
| *Use and trust* | 0.41 |
| Information source for COVID-19: Video sharing platforms (e.g. YouTube) | |
| *Not use* | 0.43 |
| *Use but distrust* | 0.50 |
| *Use and trust* | 0.65 |
| Information source for COVID-19: LINE | |
| *Not use* | 0.43 |
| *Use but distrust* | 0.57 |
| *Use and trust* | 0.56 |
| Information source for COVID-19: Twitter | |
| *Not use* | 0.44 |
| *Use but distrust* | 0.48 |
| *Use and trust* | 0.60 |
| Information source for COVID-19: Facebook | |
| *Not use* | 0.44 |
| *Use but distrust* | 0.66 |
| *Use and trust* | 0.77 |
| Information source for COVID-19: Instagram | |
| *Not use* | 0.44 |
| *Use but distrust* | 0.60 |
| *Use and trust* | 0.74 |
| Information source for COVID-19: Books | |
| *Not use* | 0.43 |
| *Use but distrust* | 0.82 |
| *Use and trust* | 0.72 |
| Information source for COVID-19: TV news | |
| *Not use* | 0.54 |
| *Use but distrust* | 0.48 |
| *Use and trust* | 0.43 |
| Information source for COVID-19: Tabloid TV shows | |
| *Not use* | 0.46 |
| *Use but distrust* | 0.43 |
| *Use and trust* | 0.46 |
| Trust in the government of Japan | |
| *Distrust* | 0.40 |
| *Trust* | 0.56 |

Full table is in STable 7.

**Table 3** shows the results of multivariable linear regression analysis with sampling weights after imputation (full table in STable 8). Lower secondary education as well as holding a master’s or doctor’s degree were associated with endorsing fewer COVID-19 conspiracy theories compared with upper secondary education (lower secondary education: B -0.089, 95%CI -0.172, -0.006; master’s or doctor’s degree: B -0.066, 95%CI -0.126, -0.006). Temporary employees (B -0.051, 95%CI -0.095, -0.007), self-employed (B -0.102, 95%CI -0.162, -0.042), unemployed or retired (B -0.078, 95%CI -0.131, -0.025), and home makers (B -0.086, 95%CI -0.135, -0.037) held fewer conspiracy theories compared to regular employees. In addition, higher household income and assets were also associated with endorsing more conspiracy theories (annual household income of ≥8 million yen: B 0.069, 95%CI 0.020, 0.117 vs. 4 to <5 million yen; household financial assets of 9 to <20 million yen: B 0.060, 95%CI 0.010, 0.109 vs. 0 to <1 million yen).

As for information sources, participants who used and trusted the websites of government agencies had fewer conspiracy beliefs than those who did not use the websites of government (B -0.109, 95%CI -0.139, -0.079). On the other hand, participants who used and trusted video sharing platforms (e.g. YouTube) (B 0.089, 95%CI 0.038, 0.139), LINE (B 0.053, 95%CI 0.009, 0.097), Facebook (B 0.089, 95%CI 0.000, 0.178), and Instagram (B 0.093, 95%CI 0.008, 0.178) endorsed more conspiracy theories than those who did not use them. Participants who trusted the information from books endorsed more conspiracy beliefs than those who did not use books (B 0.136, 95%CI 0.068, 0.204). Participants who trusted TV news endorsed fewer conspiracy theories (B -0.081, 95%CI -0.128, -0.035 vs. not used), while those who trusted tabloid TV shows held more conspiracy theories (B 0.051, 95%CI 0.015, 0.087 vs. not used).

Trust in authorities was associated with endorsing COVID-19 conspiracy beliefs. Trust in the government of Japan was associated with holding more conspiracy theories (B 0.175, 95%CI 0.139, 0.211), compared to those who distrusted government.

**Table 3. Associations between independent variables and the number of COVID-19 conspiracy beliefs**

|  | **Weighted multivariable adjusted model** | |
| --- | --- | --- |
|  | **(n=28,175)** | |
| **Variable** | **Beta** | **95% confidence interval** |
| The number of conspiracy theory beliefs regarding general vaccines | | |
| *0 (reference)* | — | — |
| *1* | 0.059 | 0.017, 0.102 |
| *2* | 0.045 | -0.004, 0.094 |
| *3* | 0.040 | -0.022, 0.103 |
| *4* | 0.178 | 0.098, 0.258 |
| *5* | 0.128 | 0.042, 0.215 |
| *6* | 0.401 | 0.299, 0.503 |
| *7* | 0.718 | 0.593, 0.843 |
| Educational attainment | |  |
| *Upper Secondary School (reference)* | — | — |
| *Lower Secondary School* | -0.089 | -0.172, -0.006 |
| *Specialised Training College (Post-Secondary Courses)* | 0.018 | -0.026, 0.061 |
| *Junior College and College of Technology* | 0.034 | -0.008, 0.077 |
| *University* | 0.009 | -0.024, 0.042 |
| *Master’s or doctor’s degrees* | -0.066 | -0.126, -0.006 |
| Employment status | |  |
| *Regular employee (reference)* | — | — |
| *Temporary employee* | -0.051 | -0.095, -0.007 |
| *Self-employed* | -0.102 | -0.162, -0.042 |
| *Employer* | 0.004 | -0.081, 0.089 |
| *Student* | -0.061 | -0.155, 0.034 |
| *Unemployed or retired* | -0.078 | -0.131, -0.025 |
| *Home maker* | -0.086 | -0.135, -0.037 |
| *Others* | -0.118 | -0.248, 0.012 |
| Annual household income | | |
| *4 to <5 million yen (reference)* | — | — |
| *0 to <2 million yen* | 0.038 | -0.024, 0.101 |
| *2 to <4 million yen* | 0.035 | -0.008, 0.078 |
| *5 to <8 million yen* | 0.016 | -0.026, 0.058 |
| *≥8 million yen* | 0.069 | 0.020, 0.117 |
| Household financial assets | | |
| *0 to <1 million yen (reference)* | — | — |
| *1 to <4 million yen* | 0.039 | -0.006, 0.084 |
| *4 to <9 million yen* | 0.020 | -0.026, 0.066 |
| *9 to <20 million yen* | 0.060 | 0.010, 0.109 |
| *≥20 million yen* | -0.016 | -0.068, 0.036 |
| Household indebtedness | |  |
| *None (reference)* | — | — |
| *>0 to <2 million yen* | -0.025 | -0.080, 0.030 |
| *≥2 million yen* | -0.016 | -0.052, 0.020 |
| Information source for COVID-19: Websites of government agencies | | |
| *Not used (reference)* | — | — |
| *Use but distrust* | 0.083 | -0.011, 0.178 |
| *Use and trust* | -0.109 | -0.139, -0.079 |
| Information source for COVID-19: Video sharing platforms (e.g. YouTube) | | |
| *Not used (reference)* | — | — |
| *Use but distrust* | -0.002 | -0.073, 0.070 |
| *Use and trust* | 0.089 | 0.038, 0.139 |
| Information source for COVID-19: LINE | | |
| *Not used (reference)* | — | — |
| *Use but distrust* | 0.064 | -0.029, 0.157 |
| *Use and trust* | 0.053 | 0.009, 0.097 |
| Information source for COVID-19: Twitter | | |
| *Not used (reference)* | — | — |
| *Use but distrust* | -0.002 | -0.069, 0.066 |
| *Use and trust* | 0.048 | -0.005, 0.102 |
| Information source for COVID-19: Facebook | | |
| *Not used (reference)* | — | — |
| *Use but distrust* | 0.042 | -0.079, 0.163 |
| *Use and trust* | 0.089 | 0.000, 0.178 |
| Information source for COVID-19: Instagram | | |
| *Not used (reference)* | — | — |
| *Use but distrust* | -0.012 | -0.120, 0.095 |
| *Use and trust* | 0.093 | 0.008, 0.178 |
| Information source for COVID-19: Books | | |
| *Not used (reference)* | — | — |
| *Use but distrust* | 0.137 | -0.011, 0.286 |
| *Use and trust* | 0.136 | 0.068, 0.204 |
| Information source for COVID-19: TV news | | |
| *Not used (reference)* | — | — |
| *Use but distrust* | -0.040 | -0.102, 0.022 |
| *Use and trust* | -0.081 | -0.128, -0.035 |
| Information source for COVID-19: Tabloid TV shows | | |
| *Not used (reference)* | — | — |
| *Use but distrust* | 0.005 | -0.043, 0.053 |
| *Use and trust* | 0.051 | 0.015, 0.087 |
| Trust in the government of Japan | | |
| *Distrust (reference)* | — | — |
| *Trust* | 0.175 | 0.139, 0.211 |

Full table is in STable 8.

Linear regression analysis with a robust error variance was used, applying sampling weights and imputation.

A positive coefficient means holding more conspiracy theories while a negative coefficient means holding fewer conspiracy theories.

The adjusted model included all independent variables simultaneously.

**5.** Additionally, recommendations for future research are missing.

**REPLY**

We have added recommendations for future research.

(Line 368–370, page 18)

Furthermore, a key area for future research is to examine the mechanisms underpinning these observations specific to Japan. This knowledge gap should be addressed to understand the phenomenon.

**Response to Reviewer 4**

This manuscript on the associations between COVID-19 conspiracy theory beliefs and sociodemographic factors, trust in government, etc. as well as with covid vaccine hesitancy tackles a relevant and complex issue.

**REPLY**

Thank you very much for reviewing our manuscript and offering valuable advice. We have addressed your comments with point-by-point responses and revised the manuscript accordingly.

**Conceptual framework**

**1.** The manuscript, however, does not include a theoretical or conceptual framework, which could significantly strengthen this manuscript.

Although not a deal breaker, it would be helpful if the authors could include a conceptual framework, perhaps with antecedents → beliefs → consequences, if not a proper theory. The inclusion of a theory or framework serves as a guiding structure for your research. Without it, the study feels somewhat exploratory or descriptive, and it becomes harder to draw clear conclusions about the mechanisms at play.

I recommend revisiting the literature review to see if there’s a theory or framework that aligns with the factors the authors are investigating. Once identified, it could frame the research questions, guide the discussion of the findings in a more structured way, and suggest avenues for future research.

**REPLY**

We have clarified our theoretical framework in the introduction section. As you pointed out, we propose our supposition: "antecedents → beliefs → consequences".

(Line 65–69, page 3)

Previous studies examined the characteristics of individuals endorsing COVID-19 conspiracy beliefs [13]. Based on previous reports, potential determinants of harboring COVID-19 conspiracy beliefs include socioeconomic and social factors, while potential consequences include health behaviours such as vaccine hesitancy and disregard for hygiene guidelines.

**Limitations**

**2.** This study relies on a “nationally representative web-based self-administered questionnaire survey.” Yet, most, but certainly not all, Japanese use the Internet and the sample is self-selected. This should be mentioned in the limitations section, including any information the authors could provide regarding who is likely left out of this “nationally representative” sample.

**REPLY**

As you pointed out, the current respondents had different characteristics from the general population in Japan. However, we used sampling weights to achieve national representativeness.

(Title)

Correlates of COVID-19 conspiracy theory beliefs in Japan: A cross-sectional study of 28,175 residents

(Line 28–31, page 2)

This study aimed to estimate the prevalence and correlates of COVID-19 conspiracy beliefs using a survey of 28,175 residents of Japan aged 16–81 years old.

**Methods:** A cross-sectional self-administered survey was conducted from September to October 2021.

(Line 111–113, page 5)

This cross-sectional study used a dataset from the Japan COVID-19 and Society Internet Survey (JACSIS) (https://jacsis-study.jp/), which is a web-based self-administered questionnaire survey.

(Line 348–355, page 17–18)

Although recruitment through an internet research agency can limit the generalizability of the findings to the Japanese population, the data include a diverse sample of residents of Japan, including a substantial representation of those with low socioeconomic status. In addition, we employed sampling weights based on census data to improve external validity. Moreover, this study explored numerous potential correlates, enabling a comprehensive assessment of the characteristics of individuals in Japan who endorse COVID-19 conspiracy theories. These strengths can enhance the generalizability of the findings.

**Consequences of correlates of COVID-19 conspiracy theory beliefs**

3. The brief description of associations between conspiracy theory beliefs and vaccine hesitancy is an important aspect of this study. I encourage the authors to give this more attention in the manuscript, including suggestions regarding future research to explore the consequences of COVID-19 conspiracy theory beliefs in addition to the correlates of such beliefs.

**REPLY**

As other reviewers have pointed out that the results section is difficult to understand, we have decided not to examine the results of consequences as the main results of our manuscript. We plan to investigate the hypothesis in detail in our next paper.
